# Monitoring and modelling of indoor environmental quality to guide indoor greening applications

**DOI:** 10.1016/j.eehl.2026.100289

**Published:** 2026-10-09

**Authors:** Prashant Kumar, Elisa Valentim Goulart, Hao Sun, John Kaiser Calautit, Karina Corada Pérez, Wenjie Song, Sandra Giulia Linnea Persiani, Kamaldeep Bhui, Douglas Booker, Brian Considine, Hossein Fatahian, Bruño Fraga, Bruno Furieri, Yuhan Huang, Peter J. Irga, Laurence Jones, Priyanka Kulshreshtha, Ashok K. Luhar, Giovanni Lo lacono, Maria Clara Vieira Martins Starling, Aonghus McNabola, Rakesh Mishra, Lidia Morawska, Zaheer A. Nasir, James O'Donnell, Hamid Omidvarborna, Christian Pfrang, Priyadarsini Rajagopalan, Neyval Costa Reis Junior, Robyn Schofield, Jane Meri Santos, Elson Silva Galvão, Divyanshu Sood, Taciana Toledo de Almeida Albuquerque, Jannis Wenk

**Affiliations:** a Global Centre for Clean Air Research (GCARE), School of Engineering, Civil and Environmental Engineering, Faculty of Engineering and Physical Sciences, University of Surrey, Guildford, GU2 7XH, United Kingdom; b Institute for Sustainability, University of Surrey, Guildford, GU2 7XH, United Kingdom; c Department of Environmental Engineering, Federal University of Espírito Santo (UFES), Vitória, 29075-910, ES, Brazil; d Department of Architecture and Built Environment, University of Nottingham, Nottingham, NG7 2RD, United Kingdom; e Sustainability Research Institute, University of East London, London, E16 2RD, United Kingdom; f Department of Architecture, TUM School of Engineering and Design, Technical University of Munich, Germany; g Department of Psychiatry and Wadham College, University of Oxford, Oxford Health NIHR BRC, Public Mental Health Implementation Centre at Royal College of Psychiatrists, Oxford, OX3 7JX, United Kingdom; h School of Civil Engineering, University of Leeds, Leeds, LS2 9LG, United Kingdom; i School of Engineering, RMIT University, Melbourne, Australia; j School of Computing and Engineering, University of Huddersfield, Huddersfield, HD1 3DH, United Kingdom; k School of Engineering, University of Birmingham, Edgbaston, B15 2TT, United Kingdom; l Centre for Green Technology, School of Civil, Environmental and Sociotechnical Engineering, University of Technology Sydney, NSW, 2007, Australia; m Plants and Environmental Quality Research Group, School of Civil, Environmental and Sociotechnical Engineering, University of Technology Sydney, NSW, 2007, Australia; n UK Centre for Ecology & Hydrology, Deiniol Road, Bangor, LL57 2UW, United Kingdom; o Liverpool Hope University, Department of Geography and Environmental Science, Hope Park, Liverpool, L16 9JD, United Kingdom; p Bhagini Nivedita College, University of Delhi, Society for Indoor Environment, India; q CSIRO Environment, Aspendale, Victoria, Australia; r School of Veterinary Medicine, Faculty of Health and Medical Sciences, University of Surrey, Guildford, GU2 7AL, United Kingdom; s Department of Sanitary and Environmental Engineering, School of Engineering, Federal University of Minas Gerais, Belo Horizonte, 31270-901, Brazil; t Queensland University of Technology, International Laboratory for Air Quality and Health, Brisbane, QLD, 4000, Australia; u Faculty of Engineering and Applied Sciences, Cranfield University, Cranfield, MK43 0AL, United Kingdom; v School of Engineering and Bernal Institute, University of Limerick, Limerick, Ireland; w School of Mechanical and Materials Engineering; UCD Energy Institute, Dublin, Ireland; x Mobile Source Laboratory Division, California Air Resources Board, Riverside, CA, 92507, USA; y School of Geography, Earth and Environmental Sciences, University of Birmingham, Edgbaston, Birmingham, B15 2TT, United Kingdom; z Post Carbon Research Centre, RMIT University, Melbourne, Australia; aa School of Geography, Earth and Atmospheric Sciences, University of Melbourne, Parkville, Australia; ab Division G - Qualitative Hydrology, Department G1 - General Water Quality Issues, Federal Institute of Hydrology (BfG), Am Mainzer Tor 1, 56068, Koblenz, Germany; ac Department of Chemical Engineering, University of Bath, Bath, BA2 7AY, United Kingdom

**Keywords:** Indoor green infrastructure, Building performance, Monitoring and modelling, Indoor-outdoor coupling, Climate change, Sustainable development goals

## Abstract

Indoor green infrastructures (iGI) can improve indoor environmental quality (IEQ), but inconsistent quantification methods undermine confidence in reported benefits and limit evidence-based design. This review synthesises monitoring and modelling methods to assess iGI impacts on IEQ, including outdoor-indoor interactions and climate change effects. Sixty studies were reviewed covering passive (potted plants, green walls, and indoor trees) and active (hydroponics, biofilters) systems. Both improve IEQ, with varying outcomes depending on configuration and environmental conditions. Passive systems, dominated by potted plants and green walls, demonstrate VOC reduction (typically 10%–30%) and help regulate temperature and humidity, while active systems achieve superior removal efficiencies for VOCs and particulate matter. The literature skews toward laboratory studies (59%) versus in-situ monitoring (33%). Many studies rely on short-duration chamber experiments (<1 m^3^; 6–48 h). Controlled settings report higher pollutant removal than real buildings, complicated by non-standardised iGI descriptors and ventilation data. Studies focus on a few plant taxa (*Chlorophytum*, *Philodendron*, *Epipremnum*, *Spathiphyllum*), measuring primarily temperature, humidity, and CO_2_. Modelling is context-specific, mainly targeting hygrothermal and CO_2_ processes in wall systems. Impacts depend on ventilation, lighting and greening intensity, with passive configurations reducing cooling demand by 22%–27% and low-light conditions weakening CO_2_ uptake [about 25 to 70 μmol/(m^2^∙s) PAR]. Outdoor–indoor interaction studies remain rare and poorly quantified. Few address future climate scenarios, creating uncertainty about benefits versus risks of elevated humidity, condensation/mould, and pollutant re-emission. Current practices inadequately attribute IEQ impacts to iGI interventions. Advancement requires standardised reporting protocols, integrated monitoring-modelling, explicit outdoor–indoor coupling, and climate-aware assessments with defined uncertainty boundaries.

## Highlights

• First synthesis linking monitoring and modelling of indoor greening.
• Short-duration chamber studies dominate: more long-term monitoring is required.
• Passive greening options can reduce VOCs by 10%–30% and cooling demand by 22%–27%.
• Combined benefits of outdoor and indoor greening are rarely quantified.
• Climate extremes and consequences for humidity/mould are rarely tested.

## Graphical abstract

**Figure:**
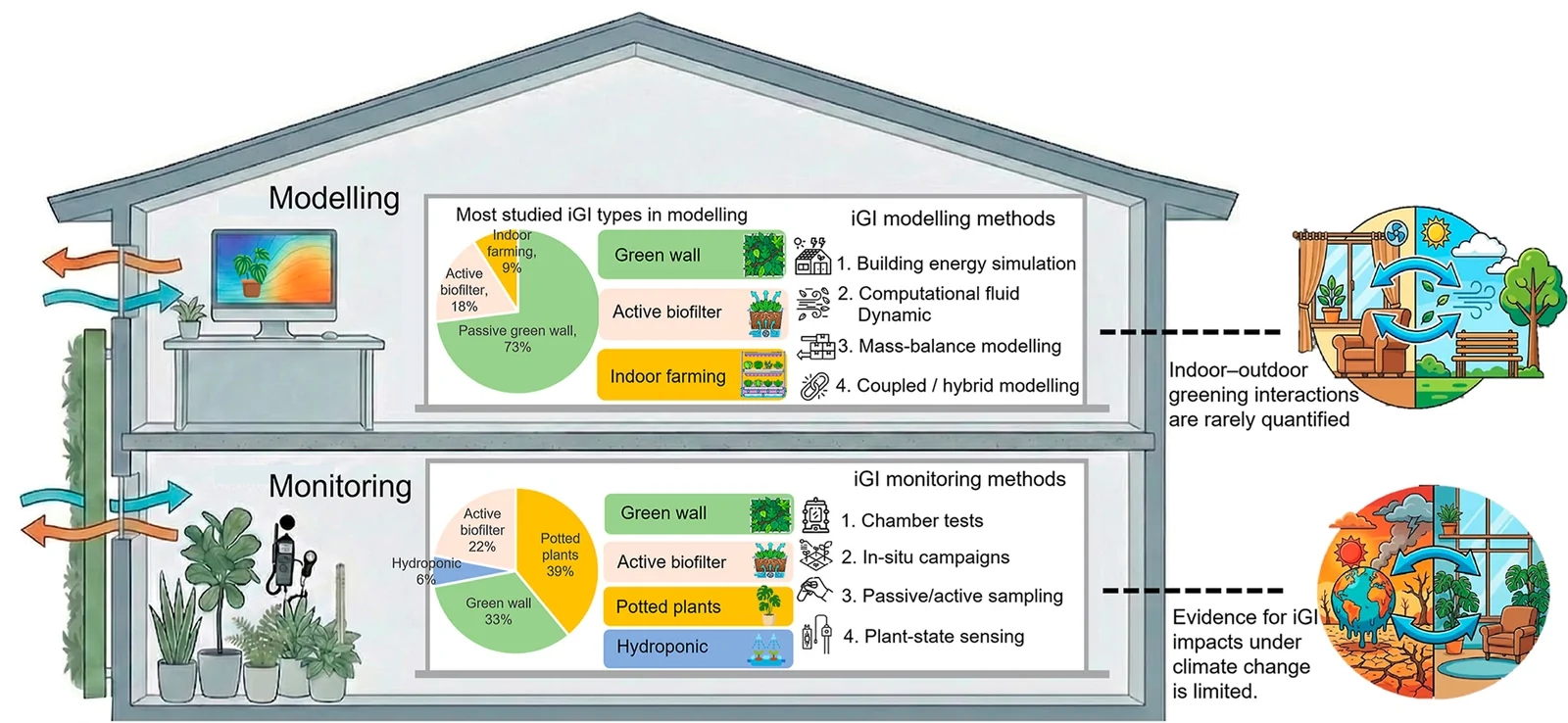

## 1 Introduction

Indoor environmental quality (IEQ) is a holistic measure of occupant health, comfort, and productivity. It is shaped by several interrelated factors, such as indoor air quality (IAQ) including the presence of bioaerosols such as bacteria, viruses, fungi, and allergens; hygrothermal conditions; and acoustic levels and lighting. People spend approximately 80%–90% of their time indoors [1], [2], [3]; however, IEQ, and particularly IAQ, has historically received less attention than outdoor air quality, despite the complex environmental exposures occurring indoors. Indoor environments can present multiple challenges for occupant wellbeing. Thermal discomfort can result from indoor temperature and relative humidity levels that fall outside occupants’ comfort ranges (typically between 18 °C and 24 °C, and RH between 40% and 60%) [4], while temporal fluctuations may further affect perceived comfort. At the same time, pollutants emitted from building materials, furnishings, cooking, and everyday activities can reach elevated indoor concentrations when emission rates exceed removal through ventilation, filtration, deposition, or chemical transformation. Where ventilation is insufficient, these pollutants may accumulate to unhealthy concentrations [5], [6]. Beyond physical conditions, limited exposure to natural elements has also been associated with psychological fatigue and declining mental well-being [7], [8].

Indoor green infrastructure (iGI) has emerged as a practical and cost-effective biophilic strategy to improve indoor environmental conditions. iGI includes intentional active or passive vegetation systems such as potted plants, indoor green walls and screens, and moss panels, grown either in conventional media or hydroponic systems [8]. Passive iGI systems rely on natural air movement, while active iGI systems employ mechanically assisted airflow to enhance air–plant interactions [9]. Indoor greening can influence enclosed environments through evapotranspiration, pollutant deposition and biofiltration, microclimate regulation, and acoustic and visual effects [8], [10]. However, despite increasing interest in iGI applications, evidence regarding their effectiveness remains fragmented and methodologically inconsistent across studies [11].

Recent developments in sustainable building standards, heightened post-pandemic attention to indoor health, and the rapid growth of green technology markets reflect growing interest in integrating indoor greening into building design and indoor environments [12], [13], [14]. To move beyond visually appealing features, reliable monitoring and robust modelling approaches are essential for establishing indoor greening as an evidence- and performance-based, standardised component of sustainable building design. However, highly heterogeneous monitoring protocols, experimental conditions, and modelling assumptions continue to limit comparability, reproducibility and large-scale generalisation across studies. Within iGI applications, “modelling” refers to a very wide spectrum of methodologies that differ greatly in their fundamental principles, primary outcomes, spatial scale, and levels of complexity. This diversity reflects the multi-scale nature of iGI processes, spanning building energy behaviour, airflow dynamics, heat and moisture transfer, and pollutant transport. Despite recent advances, there remains limited critical synthesis regarding the suitability, assumptions, validation practices, and uncertainties associated with these modelling approaches.

Decades of research on indoor plants and vertical greening systems (VGS) show growing interest in biophilic approaches to environmental control, enhancing building performance and occupant well-being. Well-designed greening systems have been associated with reductions in particulate matter (PM), improved thermal comfort, and enhanced psychological well-being [8], [10], [15], [16], [17]. However, much of the existing evidence is derived from short-term chamber experiments under highly controlled conditions that may not represent real occupied buildings.

Recent reviews have synthesised the impacts of iGI from multiple disciplinary perspectives (Table S1). Collectively, this literature highlights benefits for IAQ, particularly for volatile organic compounds (VOCs) and PM removal, with active biofilter systems frequently identified as the most effective configurations [16], [18], [19]. Psychological well-being and stress reduction constitute another major research theme. However, supporting evidence is often limited to short-term studies and relies on self-reported measures [15], [20]. Additional reported effects include humidity regulation and thermal comfort, alongside more limited evidence related to acoustics and microclimatic processes associated with plant transpiration and biological interactions [10], [21]. Despite this broad range of outcomes, existing reviews rarely interrogate how these impacts are quantified via monitoring, validated through modelling, or generalised across building types, ventilation conditions and operating scenarios.

This review addresses these gaps by critically evaluating the monitoring and modelling approaches used to evaluate iGI impacts on IEQ, rather than providing another descriptive summary of greening benefits. Particular attention is given to the methodological assumptions, validation practices, uncertainties, and applicability across building contexts. The review synthesises sensor-based monitoring, mass-balance approaches, computational fluid dynamics (CFD), building energy simulation (BES), digital twins, and hybrid or machine-learning frameworks, while identifying key research gaps related to standardisation, outdoor-indoor coupling, and climate-scenario integration. The review also examines how integrated workflows can connect empirical measurement with simulation to support more robust evaluation and decision-making under present conditions and future environmental conditions.

Our review differs from previous studies by shifting the focus from descriptive summaries of indoor and vertical greening impacts to the methodological foundations used to evaluate them. While prior studies emphasised environmental, psychological, and health benefits [10], [15], [16], conceptual and empirical findings on pollutant removal and comfort have been synthesised elsewhere [20], [21]. In contrast, this review explicitly links sensor-based monitoring protocols with a structured modelling taxonomy spanning mass-balance approaches, CFD and digital twin models, as well as hybrid methods, including machine learning. Likewise, epidemiological evidence linking greenery to mental health and biological and physiological functions has been comprehensively examined [17], [22], [23]. Our review advances the field by introducing decision criteria, uncertainty treatment, and climate-scenario coupling. These elements support scalable and predictive evaluation of iGI across different climates and under future scenarios.

The objective of this review is to critically evaluate the monitoring and modelling approaches used to quantify iGI impacts on IEQ and their applicability under current and future scenarios. Specifically, this review addresses the following research questions: (RQ1) What monitoring and modelling methods are available to assess the impacts of iGI on IEQ, and how can they be most effectively developed and deployed for decision-making? (RQ2) How do outdoor greening and environmental factors affect IEQ, and what indoor-outdoor interactions shape iGI performance and indoor exposure pathways? (RQ3) How might climate change alter the IEQ-related benefits and risks associated with iGI?

## 2 Scope, methods and outline

The scope of this review is limited to examining the influence of iGI interventions on IEQ, as assessed through monitoring and modelling under current and future climatic conditions. It covers studies analysing iGI-driven changes in pollutant removal, thermal comfort, humidity regulation, and other measurable IEQ parameters, as well as the interactions between indoor vegetation and relevant outdoor environmental factors.

To maintain conceptual clarity, several areas are excluded. Plant-production studies are omitted as they focus on growth optimisation rather than IEQ outcomes. Studies on outdoor greening alone are excluded unless they clearly link to indoor conditions. Research solely addressing psychological or well-being outcomes is not systematically reviewed unless it reports measurable IEQ mediators or directly informs indoor–outdoor exposure pathways discussed in Section 5. Finally, heating, ventilation, and air conditioning (HVAC)-only studies without vegetation are excluded. These boundaries ensure the synthesis remains focused on quantifying iGI contributions to IEQ, supporting evidence-based decision-making in building design and operation.

A systematic review following the PRISMA (Preferred Reporting Items for Systematic Reviews and Meta-analyses) guidelines [24], [25] was conducted between November 2025 and December 2025, with the final search update and conclusion completed in January 2026. Table S2 summarises the search strategy and the number of records retrieved for each keyword category using Web of Science. To capture the breadth of indoor greening research while enabling progressive filtering of a heterogeneous literature base, six conceptual keyword groups were defined. Boolean operators combined these groups to retrieve studies aligned with the review's analytical framework. Table S3 shows the combined keyword sets used to identify four thematic areas: (A) monitoring-based studies assessing iGI impacts on IEQ (RQ1); (B) modelling-based studies of iGI-IEQ relationships (RQ1); (C) studies addressing indoor–outdoor interactions relevant to iGI performance and IEQ (RQ2); and (D) studies examining climate change influences on iGI-related IEQ outcomes (RQ3).

Together, Tables S2 and S3 illustrate the contrast between the extensive literature on IEQ, monitoring, and modelling and the much smaller subset explicitly addressing indoor greening interventions. While broad keyword categories yielded large numbers of records, combining them with indoor greening typologies sharply reduced the number of eligible studies after screening.

Fig. S1 summarises the study selection process, including inclusion and exclusion criteria and reasons for exclusion at each stage. Publications were eligible if they: (i) focused on iGI interventions; (ii) were published between January 2000 and December 2025; (iii) employed monitoring or modelling to directly assess iGI effects on IEQ parameters; (iv) where applicable, examined indoor–outdoor interactions or climate-change influences relevant to iGI; and (v) were written in English. Studies were excluded if they addressed outdoor vegetation only, lacked quantitative assessment of IEQ parameters, or were non–peer-reviewed (e.g. books, theses, or technical reports). The structured screening process resulted in a core analytical corpus of 60 studies. In addition, 55 supplementary references were incorporated through citation tracking and the authors’ expert knowledge to support conceptual framing, methodological contextualisation, and broader discussion related to iGI and IEQ.

Eligible studies underwent structured data extraction covering iGI typology, experimental or building context, monitored variables (e.g. temperature, relative humidity, CO_2_, VOCs, NO_2_, PM, noise, and lighting), instrumentation, modelling approaches (e.g. CFD, building energy simulation, mass-balance models, or machine learning), and reported quantitative outcomes. Based on their primary methodology, studies were classified into two analytical groups: monitoring-based assessments (Topic A) and modelling-based assessments (Topic B). Additional contextual variables relevant to cross-study comparability were extracted where available, including chamber/room volume, ventilation conditions, pollutant loading, exposure duration, and greening density descriptors.

The review followed a four-stage selection process (Fig. S1): (1) Identification, where search strings were developed based on the study objectives and key concepts related to indoor greening, IEQ, monitoring, and modelling; (2) Screening, during which titles and abstracts were reviewed for relevance; (3) Eligibility, involving full-text assessment against predefined criteria; (4) Inclusion, resulting in a final set of 60 studies retained for qualitative and quantitative synthesis. Records focusing on unrelated topics or failing to meet the eligibility criteria were excluded.

## 3 Monitoring approaches

### 3.1 IEQ monitoring approaches for iGI impact evaluation

Fig. 1 provides an overview of global study distribution, publication trends, research methodologies, and dominant iGI types, revealing a geographically diverse but thematically convergent research landscape. Fig. 1, complemented by the keyword co-occurrence network in Fig. S2 and Text S1, reveals that IEQ research is centered on indoor air pollution characterisation and human exposure, and that a clear methodological imbalance exists, with monitoring studies dominating the evidence base as opposed to relatively few modelling studies (Section 4). Fig. 1a shows the global research coverage. European research accounts for a substantial share, including both experimental and in-situ studies focusing on living wall performance, microclimate regulation, and pollutant removal in real buildings [26], [27], [28], [29], [30]. North American research, predominantly from the United States, spans laboratory-based VOC removal experiments, green wall case studies, and the development of advanced monitoring approaches [31], [32], [33], [34]. Increasing Asian research includes studies addressing CO_2_ reduction, VOC removal, lighting effects, hydroponic systems, and integration with HVAC or air-conditioning systems [35], [36], [37], [38], [39]. Middle East research focuses on cooling effects, humidity regulation and the energy performance of green walls in workplaces and office buildings [40], [41], [42]. Australian research has focused on active botanical biofilters, particle removal, lighting optimisation, and system performance assessment [9], [43], [44], [45], [46], [47]. Fig. 1a also reveals a lack of studies from Central and South America and Africa, reflecting the critical need to develop this research field in these regions.

**Fig. 1:**
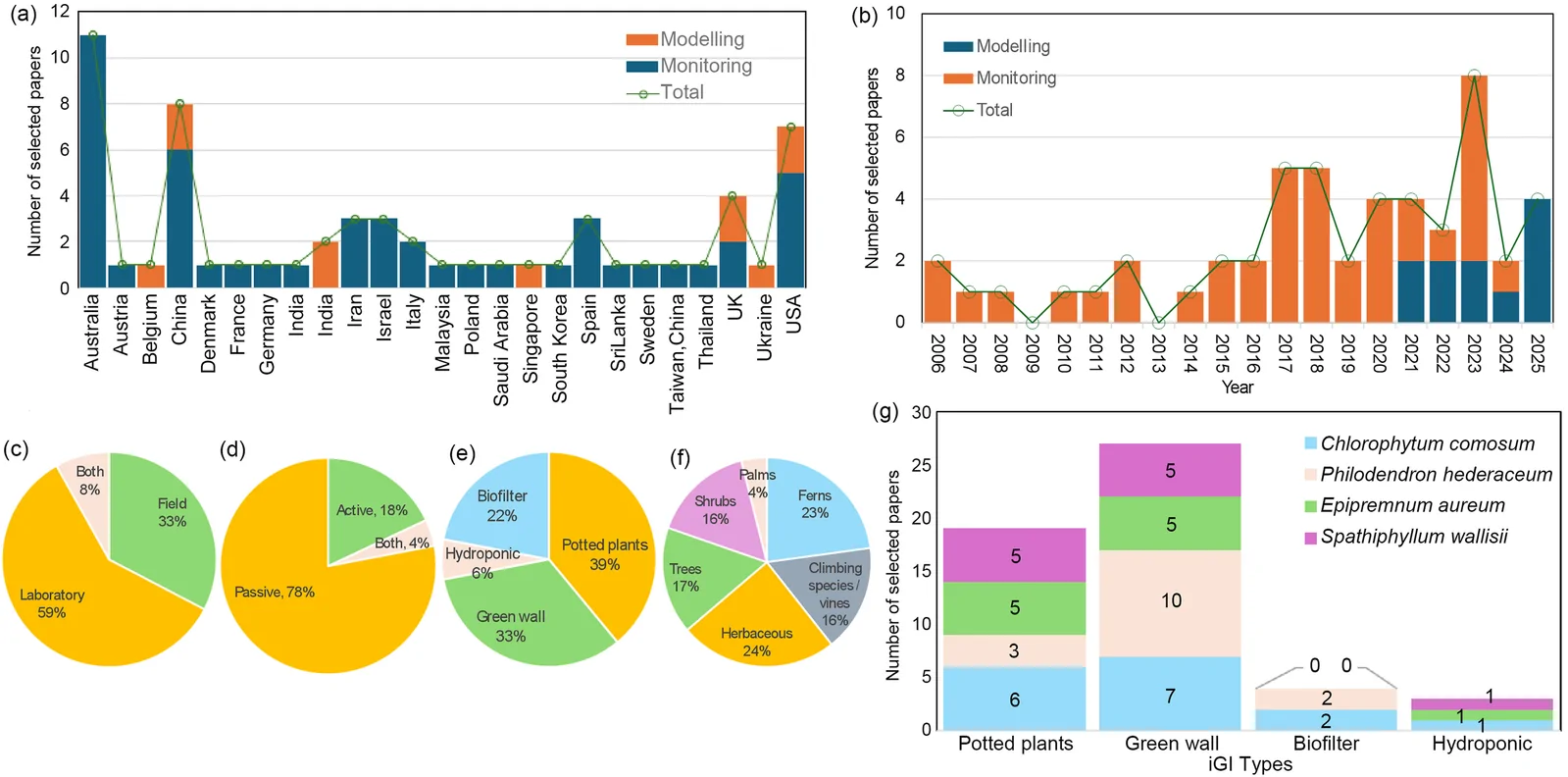
(a) Global geographical distribution of the reviewed studies on the impact of iGI on IEQ. (b) Temporal evolution of published studies over the years. (c) Methodological approaches adopted in monitoring studies. (d) System operation modes considered in monitoring. (e) Dominant iGI types assessed in monitoring studies. (f) Frequency of the most common plant functional types used in monitoring. (g) Most frequently studied plant species across the dominant iGI types in monitoring studies. iGI, indoor green infrastructure; IEQ, indoor environmental quality.

Fig. 1b shows the temporal trend of publications. Overall output remains low, but shows a modest uptick from the late 2010s onwards, with the highest annual count in 2023 (8 studies), possibly post-COVID-19 and driven by increased interest in active systems, living walls and botanical biofilters. Early work in the mid-2000s mainly consisted of laboratory-based studies using potted plants and controlled test chambers to assess VOC removal [31], [48], [49], [50]. From around 2012 onwards, research shifted towards real indoor environments, including offices, schools and circulation spaces [51], [52], [53], reflecting a move towards applied, context-specific investigations.

### 3.2 Characteristics of iGI systems

Laboratory studies dominate (59%), reflecting controlled experiments isolating plant–pollutant interactions (Fig. 1, Fig. 2). Field studies represent 33%, with 8% combining lab and field methods. Field deployments mostly occur in offices (60%), followed by classrooms (10%) and residential spaces (10%), multiple indoor settings (15%), and conferences (5%) [54], [55]. The distribution of active and passive intervention types broadly aligns with Kumar et al. [8] but shows a bias toward passive systems. Passive interventions dominate (78%), active systems account for 18%, and 4% examine both (Fig. 1d). Passive systems include potted plants (39%), passive green walls (33%), space-scale planting and hydroponic vertical farming (6%). Active systems (22%, including 4% examining both types) include mechanically ventilated biofilters and active living walls (Fig. 1e) [44], [45], [46].

**Fig. 2:**
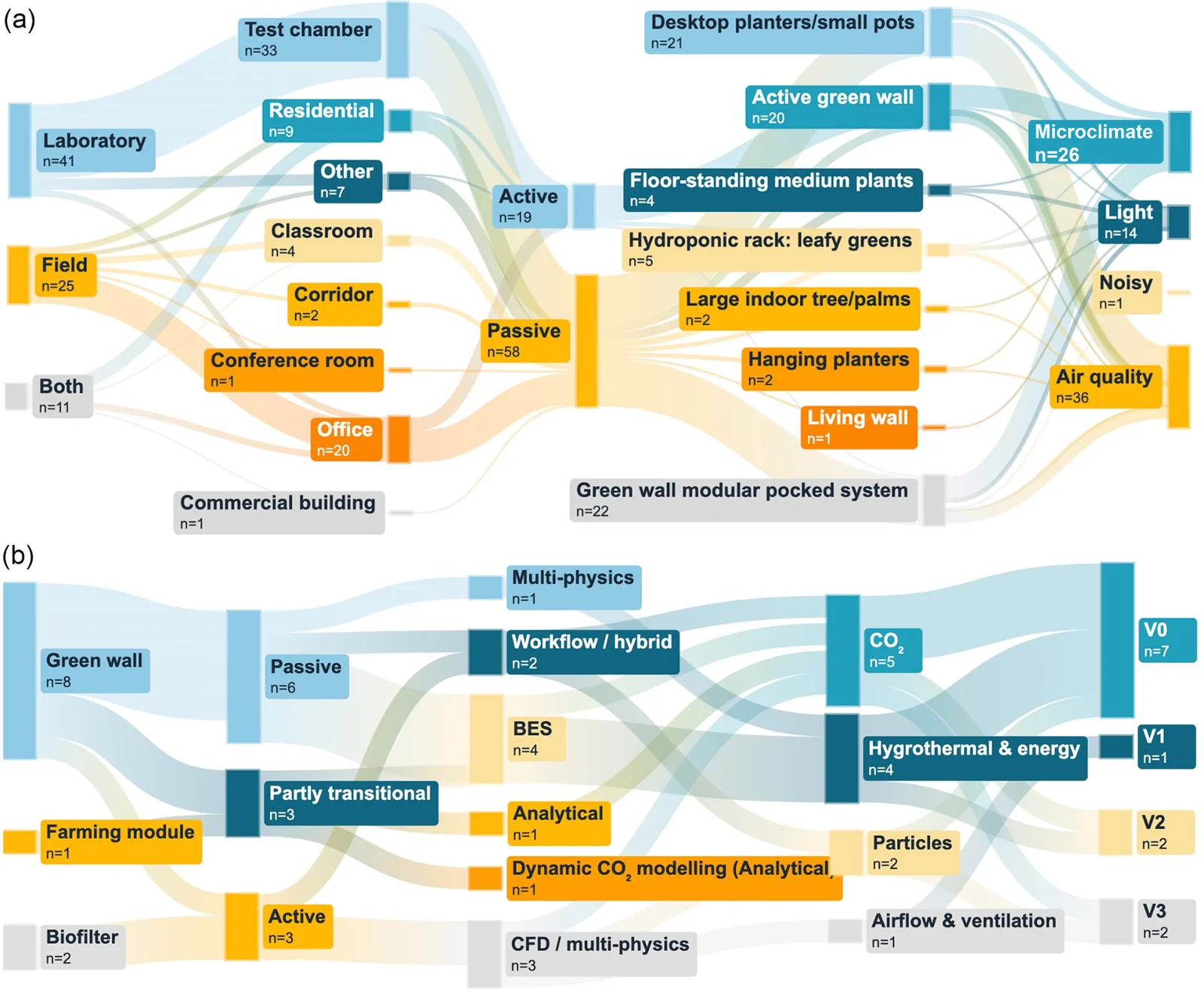
Sankey maps summarising the evidence base for iGI. (a) Monitoring studies, linking study setting and indoor space type to iGI system types and the IEQ endpoints assessed. (b) Modelling studies, linking iGI system types to modelling approaches and the target variables/endpoints simulated. Node labels report the number of studies (*n*). iGI, indoor green infrastructure; IEQ, indoor environmental quality; BES, building energy simulation; CFD, computational fluid dynamics. Validation levels: V0, no empirical validation reported; V1, limited point check or qualitative comparison; V2, measurement-informed sub-model calibration; V3, measurement-based evaluation of model outputs.

### 3.3 Plant species and functional diversity

Table S4 summarises 127 plant taxa, their frequency of use, and functional classification, based on the taxonomic resolution and common names provided in the original articles. While exhibiting substantial functional diversity (Fig. 1f), the dataset is dominated by only four herbaceous and climbing species. *Chlorophytum comosum* (spider plants; 16 studies), *Philodendron hederaceum* (heartleaf Philodendron; 15 studies), *Epipremnum aureum* (golden pothos; 11 studies), and *Spathiphyllum wallisii* (peace lily; 11 studies) constitute most of the evidence base (Fig. 1g). Ferns represent a notable proportion, mainly *Nephrolepis exaltata* (Boston fern; 6 studies), whereas woody growth forms remain underrepresented.

Studies focus on established indoor phytoremediation species, particularly *Chlorophytum comosum*, *Epipremnum aureum*, *Spathiphyllum wallisii*, and *Philodendron hederaceum*, due to their high indoor tolerance and VOC removal effectiveness [33], [38], [42], [45], [46]. Climbing species like *Ficus pumila*, *Tradescantia zebrina*, and *Philodendron scandens* were frequently used in living wall and green curtain systems, enabling dense vertical coverage and pollutant interception [26], [29], [55].

Ferns, including *Nephrolepis exaltata*, *Davallia fejeensis*, *Dryopteris erythrosora*, and *Adiantum* spp., offer high leaf surface area and transpiration capacity for humidity regulation and pollutant uptake [9], [34]. CO_2_ reduction studies incorporate robust foliage plants such as *Ficus benjamina*, *Sansevieria trifasciata*, *Aglaonema commutatum*, and *Dracaena fragrans* [30], [38]. Succulents appear mainly in experimental systems [37]. Some research explores unconventional taxa, including *Plantago asiatica* and *Taraxacum mongolicum* for formaldehyde removal [56] and *Hedera helix* for PM and VOC capture [31], [52]. Diverse plant assemblages were used to enhance structural complexity and microclimate modulation in living walls [26], [47], [57].

### 3.4 Spatial scale and monitoring duration

The studies on iGI span diverse spatial scales and monitoring durations. Chamber volumes ranged from 0.017 to 2.5 m^3^, with about 70% of laboratory studies using volumes below 1 m^3^. Small-scale chamber experiments dominate short-term assessments with volumes ranging from 0.017 m^3^ [58] to 0.324 m^3^ [38], [44], [45], and up to 2.5 m^3^ [33], [36]. Monitoring durations in these setups typically last 6–48 h, with some extending to 2–4 d [35], [52]. Intermediate-scale studies use volumes between 0.2 and 2.5 m^3^, including 2 m^3^ chambers [55] and 2.43 m^3^ cabinets [30] typically running 2–10 d or as repeated 24-h cycles over several weeks. These large differences in spatial scale, ventilation conditions, and reporting metrics complicate direct quantitative comparison of reported removal efficiencies across studies.

Field studies occur in real indoor environments such as offices and corridors. Room volumes range from 37.8 m^3^ [42] to 140 m^3^ [41]. These typically span months to a year, capturing long-term IAQ dynamics. Some studies report floor area rather than volume: 23.77 m^2^ [29], 66.6 m^2^ [57], and 146.4 m^2^ [59], limiting cross-scale comparability. The longest monitoring reported was two years [29], where a living wall was tested in a reverberation room, installed in a test building and re-tested in laboratory conditions. Another notable example is a ten-month environmental monitoring of an office space [27], underscoring interest in longitudinal evaluations. However, only a minority of studies reported key normalisation parameters such as plant leaf area density (LAD), air change rate, or greening-to-space ratios, limiting cross-study comparability and the interpretation of reported removal efficiencies.

Direct comparison of reported iGI performance across studies remains difficult because experimental configurations, chamber volumes, ventilation rates, pollutant loading, plant density, and exposure durations vary substantially. To improve interpretability and representativeness, Table S5 summarises the experimental settings reported across the reviewed studies, including room dimensions, ventilation conditions, monitoring duration and greening scale. These data enable more meaningful comparisons among studies and help distinguish highly controlled laboratory conditions from operational building environments.

It is important to emphasise that direct quantitative comparison and statistical normalisation of pollutant removal rates, temperature fluctuations, and humidity variations across the reviewed studies present significant methodological challenges (Tables S5-S8). The experimental setups identified in the literature exhibit extreme heterogeneity in several critical parameters. Firstly, the spatial scale and environmental volume vary by several orders of magnitude, ranging from micro-scale sealed chambers of only 15–375 L [35], [43], [60] to full-scale occupied environments such as offices, classrooms, laboratories, and building atriums exceeding 140–200 m^3^ [29], [33]. Secondly, ventilation conditions are highly inconsistent, including completely sealed chambers with negligible air exchange, naturally ventilated corridors and classrooms, and mechanically ventilated environments operating under widely different air change rates (ACH), ranging from approximately 0.1 to 6.0 ACH [41], [54]. Finally, the greening scale differs fundamentally across studies, varying from isolated potted plants and small modular systems to extensive active green walls exceeding 130 m^2^ in large atrium installations [33].

Because key controlling variables are not standardised across the reviewed studies, the resulting datasets remain intrinsically non-normalised. Consequently, performing a unified statistical comparison of pollutant removal efficiency, thermal regulation performance, or humidity impacts would likely produce misleading interpretations, since these performance metrics are strongly dependent on their specific environmental, operational, and experimental contexts.

### 3.5 IEQ parameters monitored

Across studies, IEQ assessment used a broad yet uneven set of indicators. Measurements focused predominantly on air quality and microclimatic parameters, particularly temperature and relative humidity. Other dimensions, like acoustic comfort and lighting conditions, received less attention. Consequently, evaluations remain skewed toward air quality performance, with IEQ's multi-dimensional nature only partially captured.

CO_2_ was consistently monitored (20 studies) in both field investigations [30], [41], [61] and chamber experiments [38], [45]. CO appeared rarely (two studies), typically within multi-gas office monitoring campaigns [49], [62]. Thermal conditions were widely reported, with temperature and RH monitored in 34 and 32 studies, respectively (Table S6). Light levels were monitored in 15 studies, reflecting their relevance for plant physiological performance and pollutant removal efficiency (Text S2).

Individual VOCs, including formaldehyde, toluene, and other aromatic compounds, were quantified in 28 studies through controlled dosing or targeted phytoremediation experiments [31], [35], [56]. Total VOCs (TVOCs) appeared in five studies, generally in field settings where aggregated pollutant loads were practical to track [33], [54]. Particulate matter (PM_2_._5_ and PM_10_) was assessed in 12 studies, frequently linked to leaf surface deposition dynamics or the biofilter filtration efficiency [44], [53], [63].

### 3.6 Quantitative performance of iGI systems

Fig. 3 summarises reported IEQ changes across iGI types, including effects on PM, CO_2_, VOCs, TVOCs, temperature and relative humidity. Across studies, both passive and active iGI can improve IEQ, but effects vary substantially depending on pollutant loading, chamber volume, ventilation conditions, greening density, plant species, and system configuration. For VOCs and TVOCs, laboratory experiments frequently report high short-term removal efficiencies, exceeding 60%–80% for toluene and formaldehyde [31], [35], [36], [46], [48], [52], [56], [64]. Field studies in offices and schools typically report TVOC reductions of 50%–75%, particularly when background concentrations exceed threshold levels [42], [49], [54], [59], [62]. Reported VOC removal efficiencies were generally higher in small-volume chambers under elevated pollutant loading, whereas field studies in occupied spaces showed lower but more stable reductions. However, several studies indicate that growth substrates alone can achieve comparable removal efficiencies and that pollutant re-emission or breakthrough may limit long-term performance, underscoring the complexity of plant–substrate–pollutant interactions [33], [46], [60]. However, substantial caution is required when extrapolating results from short-term chamber experiments to real-building applications. Many laboratory studies were conducted under highly controlled conditions using small test chambers, simplified airflow regimes, and elevated pollutant concentrations that may not represent operational indoor environments. In real buildings, iGI performance is strongly influenced by ventilation rates, occupant behaviour, spatial scale, HVAC operation, outdoor–indoor pollutant exchange, and temporal variability. Consequently, pollutant removal efficiencies and microclimatic effects reported in laboratory studies may not be directly transferable to large-scale or long-term applications without validation under real operating conditions.

**Fig. 3:**
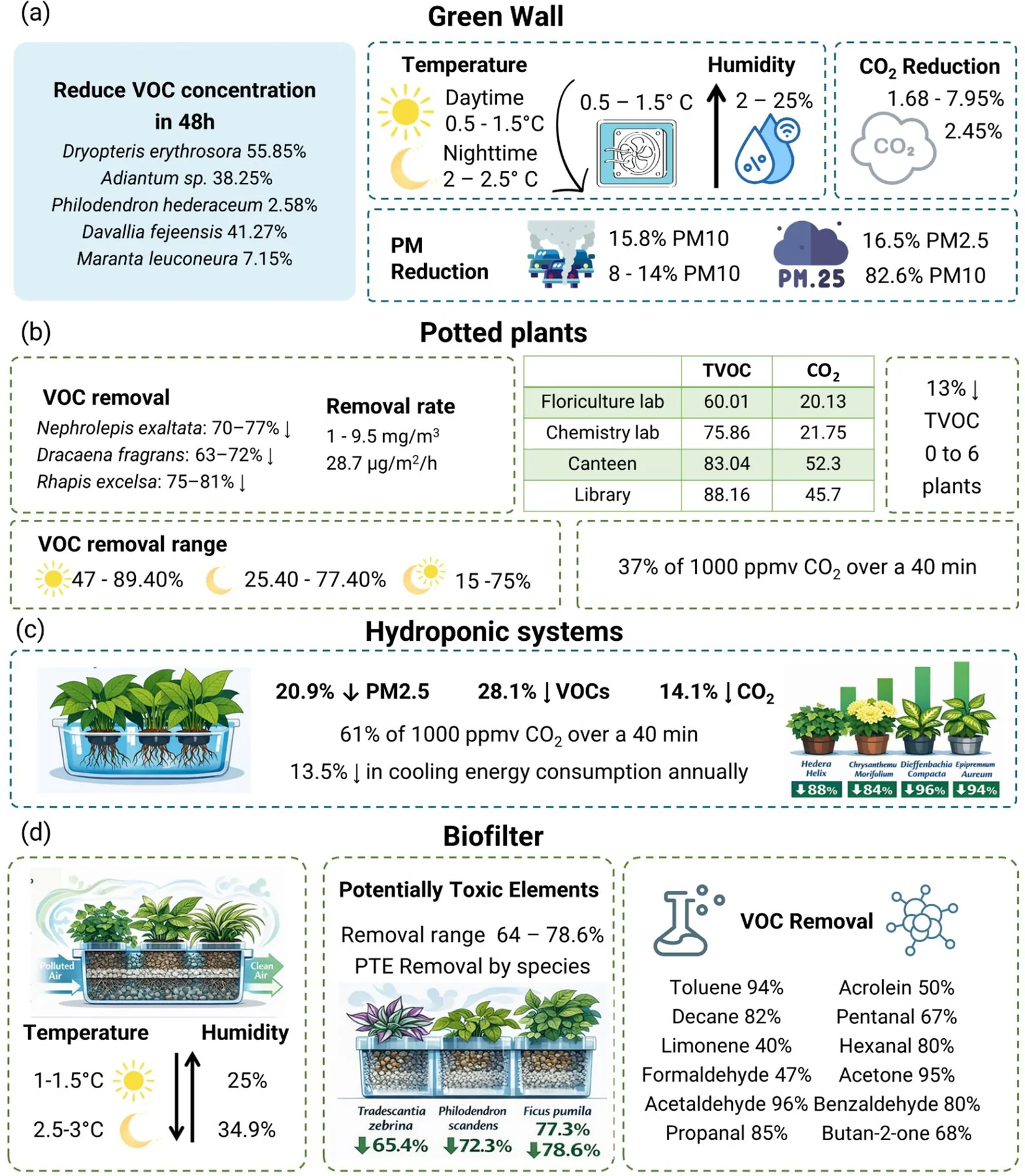
Summary of reported outcomes on IEQ changes associated with (a) green walls, (b) potted plants, (c) hydroponic systems, and (d) biofilters, including effects on PM, gaseous pollutants (CO_2_, VOCs/TVOCs), temperature, and RH. Details of the data presented on thermal parameters, gaseous pollutant removal, and PM removal can be found in Tables S6-S8, respectively.

The dataset reveals substantial variability in VOC and TVOC removal across different iGI types. Tables S6 and S7 summarise reported thermal parameters and gaseous pollutant removal, respectively. Passive planting systems exhibit consistent TVOC reduction (42%–45%, with a maximum of 78%). For individual VOCs, passive planting achieves an average reduction of 63% (range 17.5%–82.5%). Passive green walls show more variable VOC performance with median negative values of −6.02% (maximum 56%, minimum −82.21%), suggesting potential re-emission under certain conditions. Some commonly used species, including *Philodendron*, Peace Lily, English Ivy, and Chinese Evergreen, have been reported to emit or re-emit VOCs like formaldehyde and benzene under specific indoor conditions [33]. Active biofilters also achieved substantial VOC removal efficiencies under controlled experimental conditions; Torpy et al. [65] reported a mean single-pass removal efficiency of 56.6% ± 0.86% for methyl ethyl ketone (MEK), with values ranging from 55.1% to 58.5% across three trials. A single study on hydroponic vertical farming shows moderate VOC reductions, around 28% [42]. Temporal variation is also evident with daytime VOC reductions reaching 70%, compared to 44% at night [64], [66], indicating the influence of diurnal patterns.

PM and potentially toxic elements (PTE) results are summarised in Table S8. The most consistent benefits are observed for living walls and active biofilters operating under forced airflow. Active systems commonly achieve 48%–60% removal of PM_2_._5_ and PM_10_ under single-pass or steady-state configurations [44]. In contrast, long-term field deployments report broader but still substantial reductions of approximately 20%–83% across particle size fractions, following initial post-installation disturbances [42], [57]. Targeted systems designed for metal capture exhibit particularly high efficiencies, with 64%–79% removal of PTE carried by PM, largely attributable to plant uptake and retention mechanisms [55]. Evidence from leaf-surface deposition studies further supports the role of vegetation in PM capture across offices, clinical, and residential environments [53].

Evidence for CO_2_ reduction and thermal–hygrometric regulation is more modest but consistent. Chamber experiments demonstrate substantial short-term CO_2_ uptake under optimised lighting conditions, particularly in hydroponic systems and active biofilters [31], [38], [43], [45]. In real building settings, reported CO_2_ reductions are typically limited (about 2%–20%), with larger effects occurring during unoccupied periods or under low ventilation rates [27], [39], [41], [42], [61]. Across studies, living walls and biofilters are also associated with local air temperature reductions of approximately 0.5–4.5 °C and relative humidity increases of several percentage points, particularly in warm and dry conditions [9], [20], [26], [47], [58]. Additional benefits include improved acoustic performance and perceived comfort [29], [30].

Although most studies reported beneficial IEQ outcomes associated with iGI, the reviewed literature also identified several potential negative effects, operational constraints, and context-dependent limitations. Excessive increases in relative humidity were among the most frequently reported concerns, particularly in oversized or poorly ventilated systems, with some studies reporting indoor RH approaching 80%–100%, potentially contributing to thermal discomfort, condensation, mould-related risks, and even physical damage to leaves when they contact surrounding surfaces [9], [28], [30], [31]. High-moisture environments were additionally associated with increased risks of mosquito attraction, bacterial proliferation, and other pest-related concerns [39]. Several studies also reported that pollutant-removal performance may decline under unfavourable environmental conditions, including low light intensity, high ventilation rates, substrate saturation, or elevated contaminant concentrations [35], [48], [67]. Under low-light conditions, some plant systems exhibited net CO_2_ emissions due to plant and microbial respiration exceeding photosynthetic uptake [36], [43]. Other studies observed reductions in VOC removal efficiency over time due to saturation effects, while certain species were associated with transient increases in VOC or TVOC concentrations through biogenic emissions or flowering processes [33], [49], [68]. Plant physiological damage under severe pollutant exposure, including chlorophyll loss and irreversible stomatal impairment, was also reported, potentially reducing long-term pollutant-removal performance by 35%–50% during subsequent exposures [56], [64], [66]. Additional operational limitations included substrate dust dispersion, air backflow, increased pressure drop caused by dense root growth, degradation of electronic components such as light-emitting diode (LED) drivers and pumps, higher fan-energy requirements, and increased water and energy consumption associated with continuous active-system operation [42], [44], [55], [57], [58], [63]. Further information about reported negative effects and limitations can be seen in Tables S6-S8.

### 3.7 Monitoring-based iGI case studies

#### 3.7.1 Potted plants

Potted plants have been studied for their ability to improve IAQ and microclimate regulation. Research on potted plant systems is predominantly conducted in controlled laboratory settings (14 studies), with fewer investigations in real-world field conditions (3 studies) or combined approaches (2 studies). Small pots are commonly used in experiments (14 cases), while large, tree-scale potted plants are rare due to indoor space constraints. Spider plants (*Chlorophytum comosum*) are frequently tested [32], [38], with CO_2_ and VOCs being the primary pollutants studied [49], [68] using Aeroqual devices, spectrophotometers, and gas chromatography [gas chromatography-mass spectrometry (GC-MS) or GC–FID] [[31], [67]].

Laboratory studies show VOC removal efficiencies of 20%–80% depending on species, light, and exposure duration [35], [48], [50], [52], with formaldehyde reductions of 30%–70% achieved by species such as *Hedera helix*, *Plantago asiatica*, and *Taraxacum mongolicum* under dynamic fumigation [56], [64], [66]. Rhizosphere processes dominate VOC removal, while foliage contributes less [60]. Light availability critically influences CO_2_ uptake and transpiration, with meaningful reductions observed only under adequate irradiance [38].

Field studies show variable results due to ventilation and occupant behaviour. VOC and PM reductions range from 5% to 30% depending on plant density, room volume, and ventilation [49], [62], [67], with species like *Chlorophytum comosum* reducing PM by 10%–25% via surface deposition rather than metabolic uptake [53]. Microclimatic effects include RH increases of about 2%–10%, with negligible temperature changes [30].

A study by Bhargava et al. [62] on *Dypsis lutescens* potted plants demonstrated dose-dependent pollutant reduction over 16 weeks in naturally ventilated spaces. Under the specific conditions examined, increasing plant numbers significantly reduced TVOCs (up to 88%), CO_2_ (up to 52%), and CO (up to 95%), particularly in high-occupancy areas like libraries and canteens. These effects were driven by stomatal uptake, cuticular adsorption, and rhizosphere biodegradation, highlighting the importance of plant density, ventilation, and environmental factors in optimising IAQ improvements.

#### 3.7.2 Green wall

Green walls are increasingly recognised for their capacity to improve IEQ through pollutant removal, microclimate regulation, and aesthetic benefits. Most research has been conducted in field settings (9 studies), followed by laboratory experiments (6 studies), with relatively few hybrid approaches (2 studies), reflecting a growing emphasis on real-world building performance. Installations often employ diverse species assemblages (e.g. *Asparagus sprengeri*, *Chlorophytum comosum*, *Epipremnum aureum*, *Ficus pumila*, *Monstera deliciosa*, *Nephrolepis exaltata*, *Soleirolia soleirolii*, *Spathiphyllum wallisii*) to increase functional diversity [33], [40]. However, the multi-species systems complicate the attribution of pollutant removal to individual species.

Laboratory studies typically use climate chambers with controlled airflow, lighting, and automated VOC sampling, combined with advanced analytical techniques such as GC-MS [33]. Field investigations rely on portable sensor networks and multi-parameter IEQ instruments to measure temperature, RH, CO_2_, and PM under real operating conditions [9].

A case study implemented in a Turin primary school assessed VOC and PM removal by an indoor green wall. Post-installation measurements showed reduced ambient concentrations for most compounds (formaldehyde, acetaldehyde, benzene, toluene, and others), with other VOCs below detection for the entire experiment. The green wall was also reported to remove 4.42 g PM_10_ annually (0.184 g/m^2^ per year) via deposition [69]. In this context, PM removal is mainly attributed to deposition onto the expanded plant and substrate surfaces, driven by airflow-mediated transport, interception, impaction, gravitational settling, and diffusion, rather than direct metabolic uptake. Species-specific performance varied significantly, from 0.12 μg/cm^2^ (*Maranta leuconeura*) to 62.4 μg/cm^2^ (*Chlorophytum comosum*) [69]. As with most previously described in situ studies, the ambient air pollutant reductions were lower than anticipated due to structural constraints, increased natural ventilation at the time of testing (in this case COVID times) limiting optimal evaluation conditions [69].

#### 3.7.3 Hydroponic systems

Hydroponic systems (hydroculture) show considerable potential for improving IEQ through pollutant removal and microclimatic regulation. Two laboratory studies and one field study have examined their effects on PM_2_._5_, VOCs, and CO_2_. A chamber-based comparison with soil-based potted plants demonstrated higher CO_2_ removal in hydroponic systems, attributed to the absence of soil respiration and lower microbial CO_2_ generation in soilless substrates [43].

Laboratory research primarily employs test-chamber experiments to isolate the influence of growing media, root–air exchange, and airflow on pollutant removal and thermal-humidity regulation. Under these conditions, pollutant removal efficiency is typically quantified either as the percentage reduction between initial and final pollutant concentrations over a defined exposure period or through comparisons with control/reference chambers or spaces without vegetation. Using these approaches in laboratory settings, hydroponic substrates alone achieved formaldehyde removal efficiencies of approximately 39.3%–62.6% over 10 h, whereas integrated plant–root systems reported significantly higher efficiencies ranging from 81% to 96% over 24 h [31]. Furthermore, research utilizing passive hydroponic systems demonstrated that species like Syngonium podophyllum can remove up to 61% of a 1000 ppmv pulse within 40 min under enhanced lighting, while effectively depleting VOCs such as benzene at rates that outperform traditional soil-based systems for carbon drawdown. Field-based studies further reported reductions in total VOC concentrations under real operational conditions, including a 28.1% decrease measured in an occupied office environment compared to an identical non-planted control room over a 12-month period [42]. The aqueous root zone enhances pollutant uptake by promoting mass transfer and microbial activity. Additionally, passive hydroponic green walls have also been observed to lower local air temperatures by about 0.5–2 °C and increase humidity through evapotranspiration [37].

Field studies in office settings utilizing active hydroponic green wall systems report synergistic cooling and IEQ benefits, achieving indoor air temperature reductions ranging from 0.8 °C to 4.8 °C [26], [42]. Unlike traditional passive greening, these active systems facilitate an adiabatic saturation process that significantly lowers interior wall surface temperatures and mean radiant temperature [42]. In hot-arid climates where high vapour pressure deficits maximize evapotranspiration, these active systems reduce the room's sensible heat load, with a documented 13.5% reduction in annual cooling energy demand (574.5 kWh), and peak summer efficiency gains reaching 17.1%. Despite more stable pollutant removal than potted plants, field-based evidence remains limited, and system performance is sensitive to ventilation rates, occupant behavior, and operational conditions. Moreover, hydroponic installations entail higher maintenance requirements, including water quality control, nutrient management, and pump operation.

#### 3.7.4 Active biofilters

Active biofilters, typically deployed as full-height wall systems, are designed to enhance IEQ by filtering air and regulating microclimatic conditions. The majority of studies have been conducted under laboratory conditions (9 studies), with comparatively few field investigations (2 studies). Performance assessments focus primarily on the removal of PM_10_ and PM_2_._5_, particle-bound PTEs, VOCs, and on changes in air temperature and RH. Commonly studied plant species include *Tradescantia zebrina*, *Philodendron scandens*, *Ficus pumila*, *Scindapsus aureus*, *Syngonium podophyllum*, and *Chlorophytum comosum*.

Under controlled chamber conditions, active biofilters exhibit high pollutant removal efficiencies. VOC removal typically ranges from about 40% to 96% [54], depending on airflow rate, compound type, and system configuration, with ketones and aromatic compounds (e.g. MEK and toluene) achieving removal efficiencies of 56.6% ± 0.86% and 94%, respectively [44], [65]. PM removal from 45.78% for PM_0.3-0.5_ to 92.46% for PM_5-10_, strongly influenced by substrate characteristics and airflow dynamics [63]. Reductions in airborne PTEs, including metals, range from about 64% to 79% [55]. Laboratory studies also report microclimatic effects, with air temperature reductions of about 1–3 °C and increased humidity due to evapotranspiration [58].

Field studies in occupied buildings report more moderate performance, reflecting the influence of background ventilation, occupant activity and operational variability. Reductions in PM_2_._5_ and PM_10_ range from 10% to 25%, while decreases in CO_2_ and TVOC are smaller (2%–20%) [28], [39], [70]. Observed microclimatic effects include temperature reductions of 0.5–2 °C and RH increases of 3%–10%, especially under continuous operation or when integrated with HVAC systems [28], [39]. However, measurement uncertainty in field environments can complicate the direct attribution of IEQ improvements to biofilter operation alone [54].

Controlled experiments that bridge laboratory and real-world conditions report intermediate performance, with PM_2_._5_ and PM_10_ reductions of about 48%–60% [44], [63] and PTEs removal of about 65%–79% [55], largely driven by deposition and root-zone processes. Under optimised conditions, biofilters can also achieve localised temperature reductions of about 0.5–4.5 °C and increased humidity [9], [58]. While CO_2_ removal in occupied spaces is generally limited, short-term improvements have been observed under optimised lighting and airflow regimes [43], [65]. Overall, active biofilters deliver measurable IEQ benefits, particularly under controlled operating conditions.

## 4 IEQ modelling approaches for iGI impact evaluation

Eleven modelling studies linking IEQ with iGI were identified, all published after 2020 (Fig. S1). This highlights iGI–IEQ modelling as an emerging field, driven by growing interest in low-carbon passive strategies, post-COVID-19 focus on IEQ and health, and improved modelling capabilities for plant-mediated processes (Fig. 2b).

Geographically, studies originate from Europe (4), North America (2), and Asia (5). However, most studies assume temperate-climate boundary conditions and rely on generic prototype or hypothetical building archetypes, rather than being calibrated or evaluated against measurements from specific real buildings. European studies include two UK projects: a real-building atrium case and a weather-file-driven hypothetical space [71], [72]. Belgian laboratory study combining measurements with numerical modelling [73], and a theoretical CO_2_ ventilation-focused analysis from Kyiv [74]. North American research provides two EnergyPlus-based simulations using Department of Energy (DOE) prototypes across multiple climate zones [75], [76]. Asian contributions include studies from Singapore [77] and China, including an office case study in Nanjing [78] and multi-climate simulations informed by measurements collected in Guangzhou [79], and India (focusing on Azolla-based pollutant removal modelling) [80], [81]. Methodologically, all studies are simulation-led, primarily using whole BES and/or CFD, in multi-tool workflows coupling mass-balance or differential equation models with CFD, or integrating plant/green-wall sub-models into building simulation engines. While some incorporate empirical inputs such as sensor-derived CO_2_ data or calibrated parameters, only one study combines lab experiments with modelling for validation [73]. The evidence base remains largely theoretical, focusing mainly on offices (5 studies) and generic test rooms (3 studies), with no research on high-occupancy or health-sensitive environments like classrooms, hospitals, or care facilities, and few validated real-world cases.

### 4.1 iGI typology in modelling studies

Most modelling studies focus on a single iGI type (Table 1). Nearly 80% (8 of 11) model vertical wall systems, also called green walls, living walls, phytowalls, or plant-based green walls [71], [72], [73], [74], [75], [76], [77], [79]. Modelling evidence is dominated by green walls and modular biofilter systems, typically represented using idealised boundary conditions or simplified source–sink terms, whereas common potted-plant and distributed multi-pot configurations and indoor trees remain largely unmodelled compared with monitoring studies (Section 3).

**Table 1:** Overview of iGI–IEQ modelling studies (2000–2025), summarised by indoor setting, iGI types and operational mode, modelling approach and software, simulated IEQ endpoints, temporal resolution, and validation level.

Indoor setting	iGI types (typology)	Operational mode	Model paradigm	Software/platform	Key IEQ parameters	Temporal scale/timestep	Validation level (V0–V3)^a^	Ref.
Office, DOE prototype (medium office)	Living wall (vertical wall-based)	Passive	BES	EnergyPlus (IndoorLivingWall)	T, RH, Energy (cooling), PAR (driver)	Hourly transient (design-day/short-horizon reported)	V0	[75]
Residential flat (multi-room representation)	Phytowall (whole-wall)	Partly transitional	Analytical (ventilation–CO_2_)	Scilab	CO_2_ (with ΔT as buoyancy driver)	Quasi-steady, discrete wind and ΔT cases	V0	[74]
Generic/test room	Azolla biofilter	Active (in airflow path)	CFD	NR package	CO_2_, PM_2_._5_	Short window, seconds scale (about 300 s)	V0	[80]
Generic/test room	Outlet-mounted Azolla biofilter panel	Active (in ventilation pathway)	ODE decay model + CFD	NR package	Microplastics, ΔP, airflow	Minutes scale (5–30 min)	V0	[81]
Office	Living wall (vertical wall-based)	Partly transitional	BES (control-focused)	OpenStudio with EnergyPlus	CO_2_ (demand/control), ventilation demand	Minute-level transient	V2	[79]
Laboratory test assembly (wall-scale)	Passive vertical green wall	Passive	Multi-physics	COMSOL Multiphysics	T, RH	Steady-state (incl. radiative loading cases)	V3	[73]
Single-zone room (hypothetical)	Living wall (vertical wall-based)	Passive	Workflow (daylight, physiology, CO_2_)	Revit, Rhino/Grasshopper, Honeybee/Radiance, custom Python	CO_2_ (sink/source rate), PAR	Snapshot-style scenarios	V0	[72]
Atrium, real building context	Vertical greening/living wall (multi-storey)	Passive	BES + custom greening sub-model	TRNSYS coupled with MATLAB	T, RH (context), Energy (seasonal/annual)	Transient, seasonal to annual summaries	V0	[71]
Office, DOE prototype (medium office)	Hydroponic living wall (*Epipremnum aureum*)	Passive	BES	EnergyPlus (ET model embedded)	T, RH, Energy (cooling)	Hourly, design-day focus	V2	[76]
Office	Indoor farming/NFT hydroponic module (pakchoi)	Partly transitional	Dynamic CO_2_ modelling (data-supported)	NR	CO_2_	Minute-level transient	V3	[78]
Office, L-shaped layout	Plant-based green wall (flow-through)	Active (fan-assisted)	CFD	PHOENICS	Air velocity, ACH	Steady-state	V1	[77]

a Validation level: V0 = no empirical validation reported; V1 = limited point check or qualitative comparison (insufficient for system validation); V2 = measurement-informed parameterisation/calibration of a sub-model (no full-scale output validation); V3 = measurement-based evaluation of model outputs (system/component or room-scale), with explicit comparison to observed data. T, air/surface temperature (or temperature-related outputs); MP, microplastics; ΔP, pressure drop across system/component; PAR, photosynthetically active radiation (used as a plant-process driver rather than a comfort endpoint in these studies).

Passive configurations are the largest category, with several variants (Table 1). Five studies model passive vertical greening without forced airflow through the substrate [71], [72], [76]. While some incorporate empirical inputs such as sensor-derived CO_2_ data or calibrated parameters, only one study explicitly combines laboratory experiments with numerical modelling for validation [73]. The remaining study implements an indoor living wall module embedded in a whole-building simulation, again without mechanically driven flow through the planted medium [75]. By comparison, three studies model iGI as active air-treatment systems: a fan-assisted plant-based green wall and two Azolla biofilter configurations that operate within ventilation pathways [77], [80], [81]. Three studies fall into a transitional category by representing plant physiology as a time-varying source–sink driver of indoor air conditions, typically via CO_2_ balance or metabolism-based links to IEQ response, while not explicitly modelling forced airflow [74], [78], [79].

At the system level, modelling focuses on wall-integrated greening with varied botanical detail. Most studies specify vegetation at genus or species level, e.g. *Epipremnum aureum* (*Golden pothos*) [72], [76], [79], *Aglaonema roebelinii* [74], Azolla [80], [81], and pakchoi [78], while some represent vegetation functionally as diverse assemblages [71], [73]. A smaller subset uses species-agnostic parameters like LAD or prescribed transpiration rates [75], [77]. Substrate types range from hydroponic and soil-based media to mixed substrates; some studies leave substrate unspecified, modeling it implicitly via transport or resistance parameters [73], [78], [81].

Most studies adopt area-based iGI representation corresponding to whole-wall installations, but only a subset of the room dimensions, floor area, or volume is needed for normalised comparison. Reported installation scales vary substantially. For example, Jafar et al. [72] simulated a 3 m^2^ green wall within a hypothetical 48 m^2^ room, corresponding to an approximate wall-to-floor ratio of 6%. In contrast, Alsadun et al. [42] evaluated an active green wall covering 5.76 m^2^ within a 14 m^2^ office, representing approximately 41% of the room floor area. Intermediate-scale applications include the 17 m^2^ residential phytowall proposed by Mileikovskyi et al. [74], although the associated room floor area and volume were not explicitly reported. Larger architectural implementations include a 91 m^2^ living wall integrated into a multi-storey atrium environment [71]. These differences show that iGI performance metrics are difficult to compare when installation area is reported without accompanying normalisation variables such as room volume, floor area, ventilation rate, or effective foliage area.

### 4.2 Modelling approaches, configuration, and validation practices for iGI

Across the modelling studies, four broad methodological approaches emerge, spanning (i) BES, (ii) CFD and multi-physics modelling, (iii) physics-based analytical ventilation–pollutant models, and (iv) hybrid or workflow-oriented approaches, with BES and CFD being most commonly applied and increasingly combined through coupling strategies that link local flow and exchange processes to zone-scale load and control representations (Fig. 4). These reveal a clear division between building-scale performance modelling and pollutant transport and removal modelling (Table 1). (i) BES remains the dominant approach, primarily quantifying plant-driven contributions to zone heat and moisture balance and their impact on space-conditioning energy demand. Most studies adopt EnergyPlus, with some employing Transient systems simulation programs, TRNSYS-based system models [71], [75], [76]. iGI is typically treated as a modification to envelope or zone-level heat and moisture fluxes, enabling annual or seasonal energy assessments. (ii) CFD and multi-physics frameworks prioritise detailed airflow, scalar transport, and wall-scale hygrothermal behaviour. This category includes wall-assembly simulations supported by experiments, room-scale airflow and ventilation analyses, and pollutant removal modelling for biofilter systems [73], [77], [80], [81]. These methods allow fine spatial resolution but are generally limited to idealised rooms or short simulation periods. (iii) One study uses a physics-based analytical ventilation–CO_2_ model to investigate source–sink dynamics under varying wind and buoyancy conditions, sacrificing spatial detail for conceptual clarity and parametric exploration [74]. (iv) Hybrid or workflow-oriented studies link geometric and daylight modelling to plant performance and CO_2_ uptake or use simplified dynamic models to represent plant-driven indoor CO_2_ changes in indoor farming setups [72], [78].

**Fig. 4:**
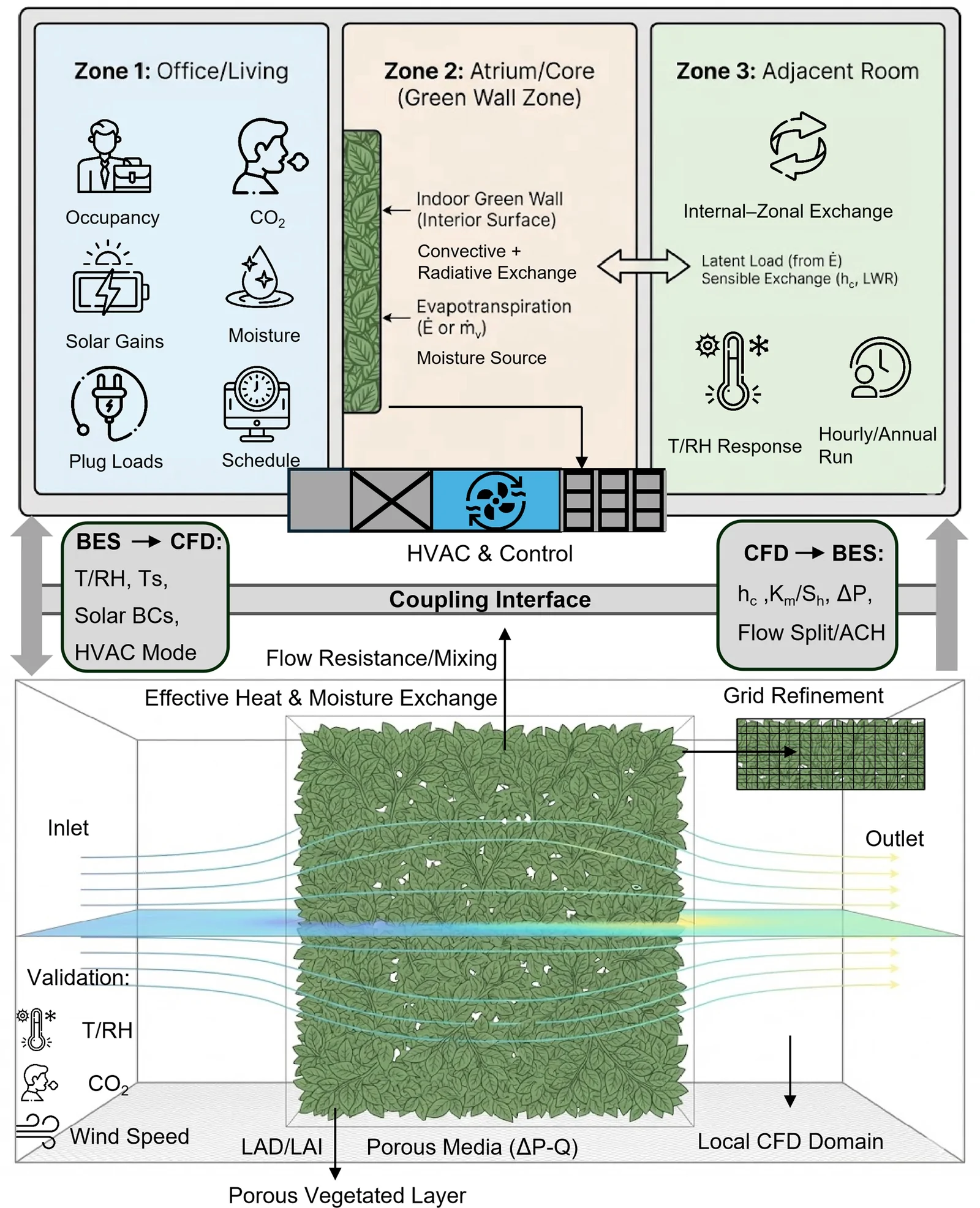
Schematic of common iGI modelling approaches. BES represents zone-scale heat and moisture balances and HVAC control to estimate energy and humidity loads, while local CFD resolves airflow patterns and transport processes around indoor greening elements. Coupled BES–CFD workflows link these scales by using CFD-derived flow and exchange parameters to inform the BES representation under realistic ventilation and operating conditions. iGI, indoor green infrastructure; BES, building energy simulation; CFD, computational fluid dynamics; LWR, long-wave radiation; T, temperature; RH, relative humidity; (Ts), surface temperature; BCs, boundary conditions; HVAC, heating, ventilation and air conditioning; ACH, air changes per hour; (Km), mass-transfer coefficient; (Sh), Sherwood number; (Hc), convective heat-transfer coefficient; (ΔP), pressure drop; (ΔP-Q), pressure drop–flow-rate relationship; LAD, leaf area density; LAI, leaf area index; (ṁᵥ), water-vapour mass flow rate; (Ė), evapotranspiration rate.

These approaches map distinct software ecosystems (Table 1). BES studies centre on EnergyPlus and OpenStudio–EnergyPlus workflows, with some coupling TRNSYS and MATLAB for bespoke vertical greening models [71], [75], [76], [79]. CFD and multi-physics studies use established solvers, applying Darcy's Law for porous media and Reynolds-Averaged Navier Stokes equations with *k-ε* turbulence closure or laminar flow assumptions [77], [80], [81]. Alvarado-Alvarado et al. [73] use a hygrothermal model for coupled heat and moisture exchange. None apply turbulence-resolving methods (e.g. large- or detached-eddy simulations) to assess turbulent mixing effects on iGI performance, even though near-wall turbulence and eddy-driven mixing can strongly govern pollutant transport, dilution, and the effective exchange between the vegetated layer and the occupied zone.

Lightweight analytical models and design-oriented toolchains improve accessibility but increase variability and reproducibility [72], [74]. CFD and multi-physics models use 3D domains, while BES and analytical methods treat indoor spaces as lumped or multizone systems, with the TRNSYS–MATLAB framework embedding a wall-normal greening sub-model within a multizone building context [71].

Three temporal modelling patterns emerge: steady-state simulations characterise airflow and wall-scale hygrothermal behaviour or support early-stage plant performance design [72], [73], [74], [77], BES studies use hourly transient simulations over design-day to annual horizons [71], [75], [76]; and fine-resolution models use second- or minute-time steps to capture CO_2_ dynamics, ventilation control, and rapid decay [78], [79], [80], [81].

Boundary conditions vary by approach. BES relies on weather files combined with zone properties, internal loads, and ventilation control strategies (e.g. infiltration and CAV/VAV or CO_2_-driven logic) to define thermal and moisture contexts [71], [75], [76], [79]. Analytical and CFD studies prescribe airflow drivers directly through inlet/outlet conditions, including wind velocity, pressure, temperature differences, and air change rates, with biofilter models adding porous-media resistance and pressure-drop constraints [74], [77], [80], [81]. iGI is commonly represented via heat and moisture fluxes, CO_2_ source–sink terms, or first-order pollutant removal coefficients. However, inconsistent reporting of wall area, room volume, and ventilation limits reproducibility and comparability [73], [76].

Simulated IEQ endpoints (Table 1) focus mainly on hygrothermal variables. Temperature metrics appear in six studies [71], [73], [74], [75], [76], [78], relative humidity in four [73], [75], [76], [78], and CO_2_ in five, modelled through ventilation mass balance, plant uptake/emission, or demand-controlled ventilation [72], [74], [78], [79], [80]. Light or photosynthetically active radiation (PAR) appears in two studies as drivers for plant behaviour [72], [75]. Airflow and ventilation proxies are included in four studies via air velocity, air change rates, flow rates, or pressure drops [74], [77], [79], [81]. Pollutant modelling beyond CO_2_ is sparse, with one study each modelling PM_2_._5_ and microplastics; no studies simulate VOCs, NOx, O_3_, CO, or noise, reflecting a focus on thermal, moisture, and CO_2_-centric framings [80], [81].

Validation practices are uneven and generally secondary to scenario exploration. Only two studies provide clear measurement-based validation: one with multi-point experimental validation of simulated temperature and humidity near a green wall [73], and another comparing minute-scale CO_2_ dynamics with monitored data in a hydroponic farming setup [78]. Two others use measurements primarily for parameterisation or calibration rather than full model validation [76], [79]. The remaining studies rely on numerical consistency checks, sensitivity analyses, or physics-based reasoning without systematic comparison to measured IEQ data, limiting confidence in model accuracy [71], [72], [74], [75], [77], [80], [81].

The integration of indoor greenery systems may also present negative trade-offs for IEQ, primarily centered on increased moisture and humidity levels. Through evapotranspiration and transpiration, these systems can raise latent cooling loads, potentially leading to RH exceeding 85%, which increases the risk of mould growth, dampness, and compromised hygrothermal comfort [75], [76]. Furthermore, indoor plants can become a net source of CO_2_ during nighttime respiration or under low-light conditions, occasionally causing concentrations to exceed 1000 ppm if natural or mechanical ventilation is insufficient [72], [74]. Operational risks also include increased heating demand in winter, thermal trade-offs from supplementary lighting, and maintenance-related biological risks or noise from fan-driven systems [71], [77], [78]. Finally, studies also highlight that certain risks, such as the emission of bioaerosols or the accumulation of VOCs and PM, remain unquantified or unresolved due to limitations in current modelling frameworks and the lack of integrated IEQ assessments [73], [80], [81].

### 4.3 Modelling-based iGI case studies

#### 4.3.1 Passive green walls for energy efficiency

Passive green walls can reduce cooling demand by moderating indoor hygrothermal conditions, but benefits can come with a humidity trade-off. Envelope-scale modelling of a modular green wall installed in front of masonry with an air cavity predicts surface temperature reductions of approximately 0.9–1.1 °C without solar radiation and up to about 10 °C under solar loading, with good agreement against climate-chamber temperature measurements [73]. At the building scale, EnergyPlus simulations using a machine-learning surrogate for hydroponic living-wall evapotranspiration report 22%–27% cooling-demand reductions in office perimeter zones across climates, yet prolonged indoor RH exceeding 85% under high greening intensity [76]. Overall, passive green walls can improve energy efficiency, but humidity management needs to be treated as a co-design requirement.

#### 4.3.2 Passive green walls for CO_2_ reduction

Passive indoor green walls can reduce indoor CO_2_, but their effectiveness depends on light levels [72]. Using a digital workflow linking daylight simulation with plant physiology, CO_2_ performance was assessed in a hypothetical room with a 3 m^2^ Epipremnum aureum wall, as detailed in Text S3. It was found that above a light threshold [about 70 μmol/(m^2^∙s) PAR], the wall reduces CO_2_, while below a threshold [about 25 μmol/(m^2^∙s) PAR], it emits CO_2_. This demonstrates that CO_2_ benefits are not guaranteed, highlighting the need for broader modelling and data to better understand these effects [72].

#### 4.3.3 Active green walls for ventilation enhancement

Active green-wall modelling often treats airflow as fixed rather than an outcome, limiting assessment of ventilation benefits. An L-shaped office with a fan-driven plant-based green wall was modelled as a flow-through element, showing ACH increased by 38%–95%, with the greatest gains near the green wall (Text S4). Air speeds mostly remained comfortable (0.01–0.3 m/s), with only localized peaks near structural features. This demonstrates that active iGI can effectively enhance ventilation without causing widespread airflow discomfort [77].

#### 4.3.4 Partly transitional green walls for CO_2_ control

Indoor green walls can help control CO_2_, but their effectiveness depends on ventilation capacity and operation. Under natural ventilation with low wind and temperature differences, a whole-wall phytowall using *Aglaonema roebelinii* acts as a daytime CO_2_ sink (about 1.56 × 10^−4^ kg/s uptake) but becomes a net CO_2_ source at night (about 1.63 × 10^−4^ kg/s release) [74]. When wind speeds fall below about 0.25 m/s, natural ventilation may fail to keep CO_2_ under 1000 ppm, showing plants cannot replace adequate air renewal. Similarly, living-wall CO_2_ uptake can delay or reduce ventilation demand in offices by about 20 min under variable air volume control, but only within CO_2_ threshold limits [79]; details are shown in Text S5. Thus, green wall performance relies on combined effects of plant metabolism, ventilation, and HVAC control, not vegetation alone.

#### 4.3.5 Active biofilters for rapid pollutant removal

Active biofilters use plants primarily to remove pollutants quickly. In one study, CO_2_ and PM_2_._5_ levels dropped by about 40% and 55%, respectively, within 5 min in a room-scale setting [80]. Another study tested a 0.25 m^2^ Azolla filter panel in a small room with mechanical ventilation, achieving particle removal rates increasing from 45% at 5 min to 85% at 30 min, with high efficiency for microplastics [81]. The panel caused a pressure drop of 0.14 Pa, and the effect of filter size on airflow resistance was also examined, showing that filter performance must balance pollutant removal with ventilation constraints [81] (Text S6). These findings show that plant-based biofilters can rapidly reduce pollutants in small settings [80], [81].

#### 4.3.6 Indoor vertical farming modules under occupancy and ventilation strategies

Indoor vertical farming modules focus on crop production, while also reducing CO_2_ and ventilation demand. An office hydroponic NFT system was studied using minute-level monitoring and modelling to capture dynamic CO_2_ changes [78]. The model treats plant CO_2_ exchange as time-varying, integrating occupancy and operation data, and is calibrated with measured CO_2_. Results showed CO_2_ levels dropped by 26%–34% compared to no plants, suggesting possible reductions in outdoor air demand and ventilation needs. This study highlights that indoor farming's effect on CO_2_ depends on how plant metabolism interacts with occupancy and ventilation, not just on having plants present.

## 5 Outdoor-indoor iGI interactions and their impact on IEQ

Outdoor and indoor greening operate as a coupled system. Vegetation mediates exchanges of energy, moisture, pollutants, sound, light, biological agents and sensory cues between urban environments and indoor spaces. Outdoor trees, parks and VGS modify façade boundary conditions, microclimate, pollutant fields, noise and visual context through radiative, convective, evaporative and biological processes. These altered conditions are then transmitted indoors via the building envelope and ventilation, which act as selective filters shaped by construction, operation and occupant behaviour. iGI further modifies thermal, chemical, acoustic and psycho-physiological exposure. Together, outdoor and indoor greening enable layered regulation of IEQ from the urban scale to the occupant scale (Fig. 5).

**Fig. 5:**
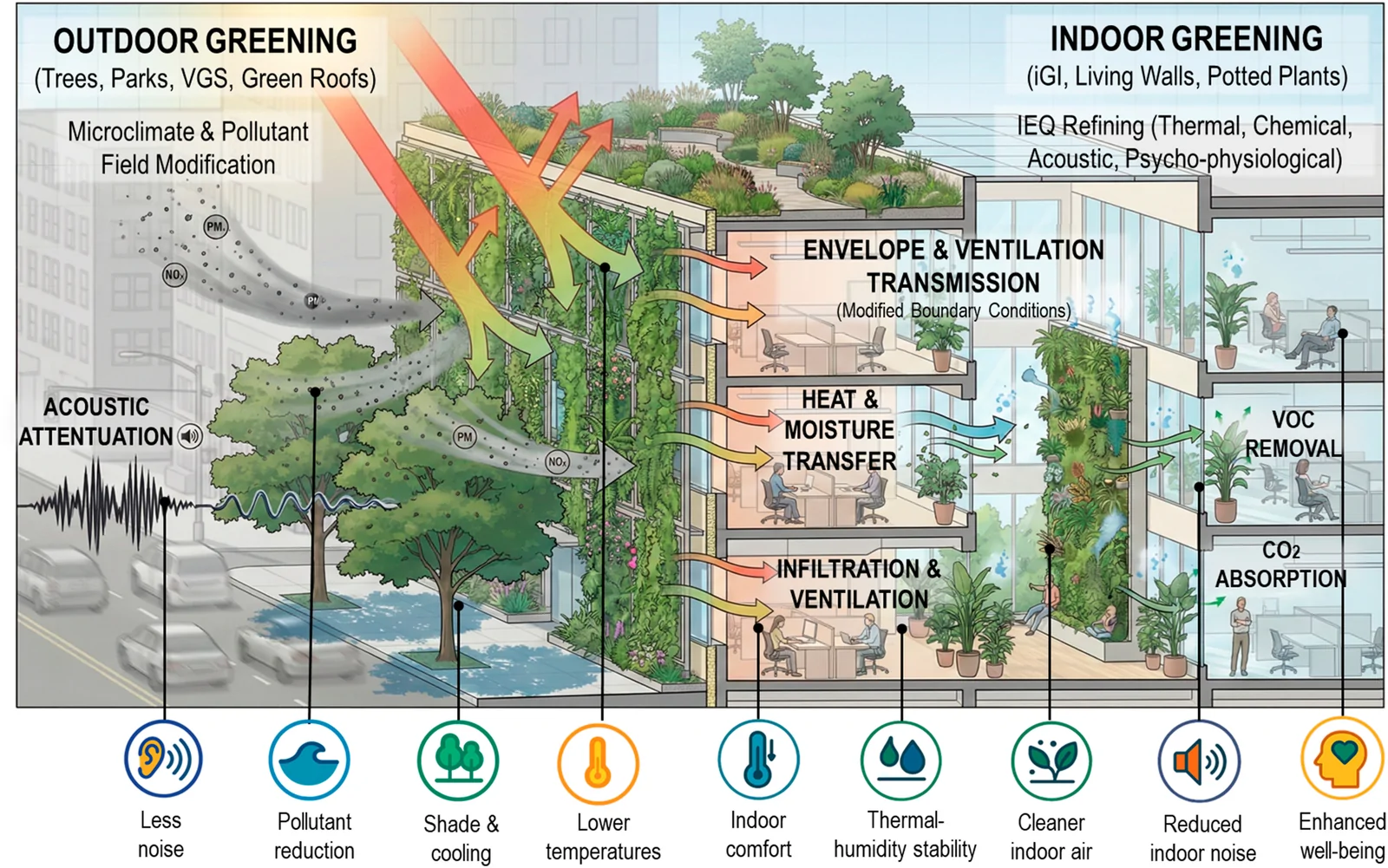
Schematic illustration of how integrated indoor-outdoor green infrastructure modifies the urban microclimate and influences IEQ. These modifications interact with building facades and ventilation systems to affect the transfer of heat, moisture, and pollutants indoors. In parallel, indoor greening contributes directly to IEQ through thermal regulation, chemical (VOC/CO_2_) absorption, acoustic attenuation, and psycho-physiological benefits.

This section synthesises outdoor–indoor pathways identified through monitoring (Section 3) and modelling studies (Section 4), and highlights key knowledge gaps and research priorities. A systematic search identified only seven relevant studies (Fig. S1); these are summarised in Table S9 together with additional studies identified through cross-citations and expert knowledge. In parallel, indoor greening contributes directly to IEQ through thermal regulation, chemical (VOC/CO_2_) absorption, acoustic attenuation, and psycho-physiological benefits.

### 5.1 Impact of outdoor greening and environmental factors on IEQ and iGI interaction

#### 5.1.1 Microclimate modification and heat–moisture dynamics

**Façade-scale vertical greening.** Green façades and living walls have been reported to modify outdoor–indoor heat and moisture transfer by altering façade boundary conditions through shading, evapotranspiration, thermal resistance and moisture buffering [82]. VGS are typically modelled as additional façade layers that integrate shading, evapotranspiration, thermal mass and moisture buffering, thereby modifying conductive heat flow and vapour diffusion. Their performance depends on outdoor forcing (e.g., solar radiation, wind, rainfall, air temperature), which governs plant physiological responses (e.g., leaf temperature, stomatal conductance, substrate moisture) and feeds back into indoor temperature and humidity. Rugani et al. [83] combined field monitoring of a green façade on a research building in Rome with a validated dynamic grey-box model. Across simulated summer conditions and multiple wall assemblies, the green wall reduced thermal-wave amplitude by 24%–39% and incoming heat flux by 35%–48%, with peak indoor air temperature reductions up to 5.2 °C, greatest for lightweight or low-performing walls. In their framework, the VGS acts primarily as a boundary-condition modifier, lowering effective external thermal forcing (akin to a reduced sol–air temperature), and aggregating shading and vegetation-related effects. Field evidence across climates is broadly consistent. In Guangzhou, China, vertical greening reduced exterior wall surface temperatures by 8–10 °C and indoor temperatures by 1–3 °C [84]. Studies in Colombo, Sri Lanka, report comparable exterior cooling (up to 8 °C), modest RH increases (1.6%–1.8%) and indoor temperatures maintained near 28 °C [85]. Complementary modelling in Catania, Italy, using dynamic TRNSYS simulations shows that VGS can lower peak inner-surface temperatures by 1–2 °C and reduce peak heat flux, again with larger benefits for lightweight walls [86]. In warm–humid contexts, moisture effects become more critical: Baghaei Daemei et al. [87] found that an ivy-covered north façade in Rasht (Iran) reduced average indoor air temperature by up to 1.1 °C and indoor RH by 32% (relative to a bare wall). By contrast, Widiastuti et al. [88] reported that while increased leaf coverage reduced temperatures, green façades raised indoor RH (up to 72.5%), increasing effective temperature relative to a bare wall. These contrasting outcomes highlight the need for humidity-sensitive comfort metrics.

**Trees, courtyards and neighbourhood greening.** At larger scales, trees and ground vegetation cool façades and indoor spaces primarily through shading and evapotranspiration, while courtyards and street canyons act as climatic “filters” that condition radiative and air loads before they reach the building envelope. Pastore et al. [89] coupled ENVI-met with EnergyPlus and reported indoor temperature reductions of up to 4.8 °C when neighbourhood greening was combined with building retrofits. Taleghani et al. [90] observed local outdoor cooling of 3.6–3.8 °C and an average indoor air-temperature reduction of 2.9 °C under an “additional trees” scenario during a heatwave. Courtyard studies [91], [92] demonstrate strong sensitivity to tree species, configuration (deciduous vs. coniferous) and orientation. In summer, rooms facing shaded courtyards experience substantial indoor cooling, up to a 4.3 °C reduction in operative temperature [91] and up to 6 °C reduction in air temperature [92]. In winter, however, shading from evergreen tree species can increase heating demand in some zones, highlighting seasonal trade-offs and the importance of careful species selection.

**Green roofs and combined roof–façade systems.** Combining vegetated envelope elements enhances buffering capacity. Vegetation functions as a distributed thermal–moisture reservoir that damps diurnal and seasonal fluctuations through cumulative shading, insulation and evapotranspiration. Twin-room experiments in Xiangtan, China [93], [94] show that combined green roofs and vertical greenery reduce operative temperature peaks by 1–2 °C, halve roof surface temperature swings, and either reduce summer cooling energy use by 7%–8% at equivalent comfort (via higher set-points) or decrease spring discomfort hours by 15%–28% in free-running rooms by stabilising both dry-bulb temperature and RH.

**Interaction with indoor greening.** Outdoor greening primarily sets boundary conditions (thermal and moisture loads) within which indoor plants, living walls and biofilters operate, especially in naturally ventilated or mixed-mode buildings where latent loads are weakly controlled. Among the reviewed studies, the case in Changzhou, China [59] is one of the few to instrument both layers. An external west-façade VGS reduced indoor temperature amplitude and increased thermal inertia (attenuation +19.5%, delay +41%), while indoor greening in the atrium produced the lowest RH variability among all monitored spaces, without clear additional air-temperature reductions. This pronounced RH stabilisation, greater than in mechanically conditioned offices, supports a sequential buffering interpretation: outdoor greening attenuates envelope loads, while indoor greening fine-tunes humidity and local microclimate. Explicit physical coupling between these layers, however, remains rarely modelled.

#### 5.1.2 Pollutant deposition, transformation and transport

**Outdoor greenness and indoor exposure.** Epidemiological studies indicate that outdoor greenness can lower pollutant concentrations at the building boundary and, through infiltration, indoors. Indoor–outdoor exchange is increasingly conceptualised as a flow network in which vegetation acts both as a passive sink and a biologically active transformation interface. In Barcelona, Spain, Dadvand et al. [95] found that higher normalised difference vegetation index (NDVI) around the homes of pregnant women was associated with lower outdoor and indoor PM_2_._5_ concentration, as well as substantially lower personal exposure, after controlling for background pollution and time–activity patterns. These effects were attributed to dry deposition on vegetation and modified dispersion. Similarly, Matthaios et al. [96] showed that inner-city schools with higher NDVI (within 270–1230 m) and greater distance from major roads had markedly lower indoor traffic-related PM_2_._5_, NO_2_ and black carbon, even after adjusting for outdoor pollution, meteorology, and ventilation type.

**Façade-scale PM removal.** Field measurements on vertical green façades in temperate climates report modest reductions ranging between 1 and 2 μg/m^3^ in PM_2_._5_–PM_10_ directly in front of densely vegetated façades compared with bare walls. Effectiveness depends strongly on foliage thickness, coverage and plant health [97]. Removal mechanisms include surface deposition, aerodynamic effects and potential stomatal uptake. Indoors, studies of living walls in Changzhou, China show that an indoor VGS with potted plants reduced PM_2_._5_ and PM_10_ by 16%–21% and halved formaldehyde levels relative to a comparable hall without vegetation, despite similar outdoor PM conditions [59].

**Combined outdoor–indoor filter chain.** A review of 73 outdoor and indoor VGS studies by Fonseca et al. [15] concludes that green façades and living walls can reduce PM and gaseous pollutants at both street-canyon and room scales, but are typically examined in isolation. When considered together, these systems form a two-stage pollutant mitigation chain: outdoor vertical greenery reduces exposure at the façade and immediate surroundings, while indoor systems remove residual VOCs and PM from recirculated indoor air. This constitutes a biologically mediated transformation across the indoor–outdoor boundary, yet very few studies explicitly quantify the cumulative effect from street level to the indoor breathing zone.

#### 5.1.3 Noise, acoustics and light

Beyond thermal comfort and IAQ, vegetation also alters acoustic and lighting conditions. Vegetated systems behave as soft, porous layers that absorb and scatter sound. Azkorra et al. [98] reported that a modular green wall achieved mid-to high-frequency absorption (α_w = 0.40) and a modest improvement in airborne insulation (Rw = 15–18 dB), primarily due to the porous substrate. Tang et al. [99] found that sedum-based “paving green walls” reduced sound pressure levels by 7–16 dB, depending on initial noise levels, while also lowering wall-surface temperatures. At the street canyon scale, vertical greening can reduce façade sound levels by several decibels, particularly when applied to upper walls or roof edges and combined with air cavities that enhance low-frequency absorption; however, most evidence derives from laboratory studies and simplified outdoor models rather than long-term *in-situ* measurements [100].

Regarding lighting, trees, green façades, and courtyard vegetation reduce solar irradiance and wall temperatures but can also limit daylight penetration [101], [102], highlighting the need to balance shading benefits with visual and daylighting requirements.

### 5.2 Revealing indoor–outdoor interactions via iGI monitoring and modelling

Studies that explicitly integrate both outdoor and indoor greening remain scarce (Fig. S1). Nevertheless, existing monitoring (Section 3) and modelling (Section 4) approaches provide a framework to analyse their interactions (Fig. 6).

**Fig. 6:**
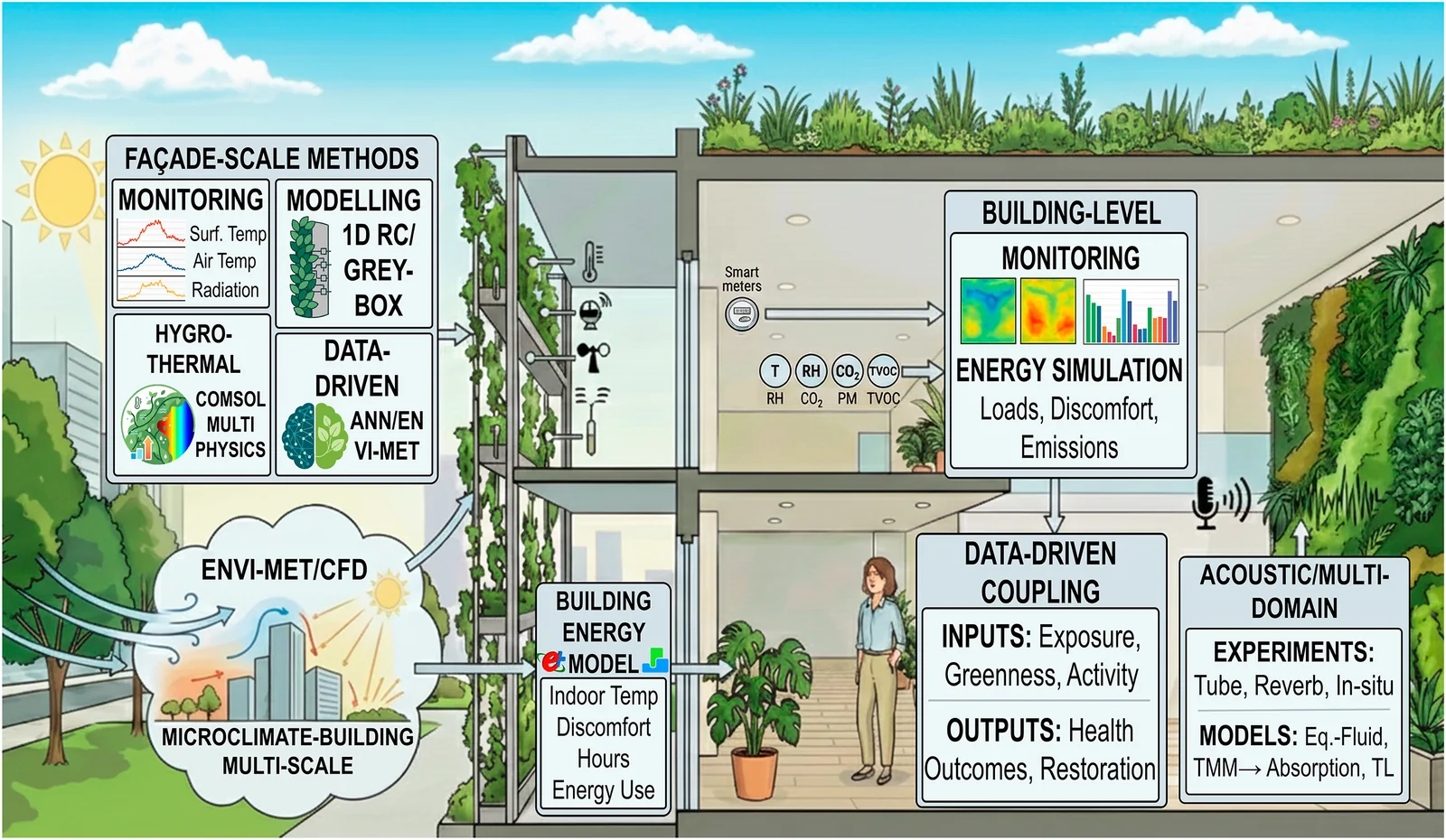
A schematic illustration of a multi-scale monitoring and modelling framework for outdoor–indoor greening and IEQ. The framework integrates hygrothermal, microclimate, and energy simulations with IEQ metrics (thermal comfort, air quality, and acoustics) and data-driven methods to assess energy performance, human exposure, and health and well-being outcomes. IEQ, indoor environmental quality; ANN, artificial neural network; ENVI-met, three-dimensional microclimate model; TL, transmission loss; TMM, transfer matrix method.

#### 5.2.1 Façade-scale monitoring and grey-box/hygrothermal modelling

Early façade-scale studies inferred the effects of VGS by monitoring façade surface temperatures and cooling electricity use, reporting clear reductions in wall temperatures and summer cooling demand [103]. Building on this, Rugani et al. [83] adopted a façade-to-room approach using a multi-year sensor network (capturing outdoor air and soil–air temperature, solar radiation, wind, leaf and soil temperatures, and indoor air temperature) combined with a 1D RC grey-box model. Measured sol–air conditions, with and without vegetation, were applied as boundary conditions to estimate indoor temperatures and wall heat flux across wall types and years. This approach yields design-ready coefficients (attenuation factor *AF*, Green Factor *K*v, phase-shift factor *t*) that can be integrated in building energy tools without explicitly resolving vegetation physics.

Alvarado-Alvarado et al. [73] extended this line of work using a controlled-climate 3D hygrothermal COMSOL model to resolve coupled heat and moisture transfer through vegetation, substrate, air cavity and wall. Their results attribute masonry surface cooling mainly to the air cavity (about 60%), with evapotranspiration (30%–48%) and insulation accounting for the remainder, underscoring cavity design and leaf-area density or water saturation as key design parameters. In humid subtropical conditions, Baghaei Daemei et al. [87] combined field monitoring with EnergyPlus, Artificial Neural Network (ANN), and ENVI-met simulations, showing that a north-facing ivy wall provides modest indoor cooling but substantially reduces indoor RH compared to a bare wall; ANN surrogates enabled rapid predictions based on outdoor temperature, RH and façade type. Similarly, Nocera et al. [86] employed a calibrated TRNSYS façade–zone model to demonstrate that VGS can reduce inner surface temperatures by up to 2 °C and peak heat flux by up to 60% in lightweight walls, confirming that shading, radiative exchange and evapotranspiration significantly modify façade boundary conditions.

#### 5.2.2 Multi-scale microclimate–building coupling

Several studies couple microclimate models (ENVI-met or other CFD models) with building energy models to capture vegetation effects from the neighbourhood to the indoor scale [89], [90], [104]. Typically, ENVI-met is used to simulate how trees, hedges, grass, and building-integrated vegetation alter local air temperature, wind and radiative exchange, and these outputs are then translated into custom weather files or boundary conditions for EnergyPlus or DesignBuilder, which predict indoor temperatures, discomfort hours, and, in some cases, energy demand. Results consistently indicate that outdoor vegetation can be a dominant driver of indoor cooling in social housing and courtyard buildings. When combined with window upgrades, green roofs and living walls, greening strategies can deliver 4–5 °C indoor cooling on hot days, while also revealing potential winter heating penalties and strong sensitivity to orientation, urban form and ventilation. Courtyard-focused studies [91], [92] further integrate validated CFD with porous vegetation models to capture both aerodynamic effects (ventilation and sheltering) and evapotranspiration cooling. These studies report indoor temperature reductions up to 6–7 °C in favourable rooms, although benefits are highly spatially heterogeneous and may be insufficient under extreme heat conditions.

#### 5.2.3 Building-level monitoring and energy simulation

Building-scale studies [85], [93], [94], [105], [106], [107] integrate façade or roof surface temperature monitoring, IEQ measurements, and energy simulation to evaluate the performance of green roofs, façades and courtyards. Typical methodologies combine parallel measurements on vegetated and reference surfaces with EnergyPlus, DesignBuilder, or TRNSYS simulations of thermal loads, discomfort, and energy use, benchmarked against metered data. Across studies, simplified modelling approaches frequently underestimate greening benefits, largely due to the omission of evapotranspiration effects and occupant behaviour. The Changzhou case study [59] advances this approach through long-term indoor–outdoor monitoring (temperature, RH, PM, CO_2_, TVOCs, formaldehyde) in spaces with and without external and indoor VGS. By linking greening strategies to thermal comfort [predicted mean vote (PMV) and predicted percentage of dissatisfied (PPD), thermal inertia], IAQ, and annual electricity bills, the study provides a rare whole-building perspective on IEQ and energy performance.

#### 5.2.4 Data-driven and statistical coupling of greenness and exposure

At the exposure and health interface, several studies adopt statistical rather than physics-based frameworks, combining (1) indoor or personal exposure indicators (e.g. PM_2_._5_, NO_2_, black carbon) or mental-health outcomes, (2) greenness metrics (NDVI buffers, green-view indices, or perceived greenness), and (3) time-activity diaries with building and ventilation covariates [95], [96], [108]. These approaches infer causal pathways whereby outdoor greenness reduces ambient concentrations or alters activity patterns, leading to lower indoor or personal exposure, or whereby indoor and outdoor greenness enhances perceived restorativeness and reduces loneliness or depression. A parallel psycho-physiological and behavioural evidence base exists, often relying on NDVI/self-report and rarely measuring physical IEQ mediators (Text S7). While not resolving physical mechanisms, these studies provide transferable methodological templates (such as mixed-effects and structural equation models) that could be strengthened by incorporating sensor-based IEQ mediators.

#### 5.2.5 Acoustic and multi-domain monitoring

In the acoustic domain, façade-scale experiments [98], [99] and recent reviews [100] combine laboratory measurements (impedance-tube and reverberation-room tests) with *in-situ* façade monitoring and equivalent-fluid or transfer-matrix models to quantify sound absorption and transmission loss in VGS. Although these results are rarely propagated into whole-room or building-scale acoustic simulations, they yield boundary-condition parameters and design-relevant insights, such as the influence of substrate porosity, air cavities, leaf-area density, and canyon placement, that could be integrated into multi-domain IEQ modelling alongside thermal and air-quality effects.

## 6 Climate change impacts on indoor greening benefits for IEQ

Climate change is reshaping both outdoor environmental conditions and the indoor systems that respond to them. Rising temperatures, more frequent and intense heatwaves, shifts in humidity regimes, and evolving outdoor pollutant patterns directly affect how vegetation interacts with indoor environments. The performance of indoor greening systems is therefore highly climate-dependent, as their cooling, humidifying, and pollutant-removal functions rely on temperature and vapour-pressure gradients, stomatal conductance, radiation, airflow, and leaf-surface deposition processes [8], [94], [109]. Building on the outdoor–façade/ventilation–indoor pathways discussed in Section 5, climate change primarily alters outdoor microclimates and pollutant fields, thereby modifying façade and ventilation boundary conditions for heat, moisture, and pollutants. These shifts influence infiltration and ventilation loads, ultimately redefining when indoor greening enhances IEQ versus when it may introduce moisture-related or air quality risks (Fig. S3).

As climate change intensifies, through hotter summers in temperate regions, increased humidity in tropical zones, and warmer winters in cold climates, the benefits of indoor greening are unlikely to remain constant (Table 2). Depending on the climate context, greening may enhance cooling and pollutant removal, or conversely introduce moisture-related risks, higher maintenance demands, and pollutant re-emission. For example, a plant may reduce indoor temperatures by 0.5–2 °C in temperate climates but by 3–6 °C in hot-arid regions, where larger thermal gradients and stronger evaporative potential amplify cooling [82]. These gains, however, are often accompanied by increased irrigation and water demand [82], underscoring the need to balance IEQ benefits with operational resource constraints in climate-resilient design.

**Table 2:** Climate-change effects on iGI benefits for IEQ across climate contexts, summarising expected performance shifts, underlying drivers, and design and operational implications.

Climate context	How climate change modifies the IEQ benefits of indoor greening	Why the effect changes	Implications for design and operation
Hot-arid/desert (e.g., Saudi Arabia)	Higher ambient temperatures enhance evapotranspiration-driven cooling (night-time drops of 12–21 °C) [110]. However, plant water demand increases and indoor RH may rise, creating potential mould risks.	Expansion of hot-arid zones and longer heatwaves increase sensible heat gradients and intensify plant latent heat flux [111], [112].	• Select drought-tolerant species with high cooling efficiency. •Integrate dehumidification or night-purge ventilation in peak season. •Use adaptive airflow control (variable fan speed).
Hot-humid/tropical-subtropical (e.g., Delhi, Singapore)	Sensible cooling from vegetation is reduced, and latent moisture release becomes dominant, pushing indoor humidity toward saturation and increasing condensation risk.	Warmer, more humid futures (higher specific humidity) reduce vapour-pressure deficits, limiting sensible cooling while increasing moisture load [111].	•Prioritise shading-based greening over moisture-intensive living walls. •Use moisture-driven irrigation and local exhaust ventilation. •Choose low-transpiration species for interior zones.
Temperate/mixed-climate (e.g., Europe, North America)	Summer cooling benefits increase under more frequent heatwaves, while milder winters reduce the value of wintertime heat gains from vegetated façades.	Reduced heating degree days + increased cooling degree days shift the seasonal balance; longer warm seasons improve summer performance [112].	•Use dynamic glazing or polycarbonate panels whose thermal conductance can be switched according to season. •Design operable or dual-mode façade systems (open for summer cooling, closed for winter solar gain).
Cold-arctic (e.g., Iqaluit, Cambridge Bay)	Indoor greening contributes more to comfort and daylighting than thermal control. Warmer winters slightly increase the relative value of greenhouse-type greening but raise risks of condensation during freeze–thaw cycles.	Arctic warming reduces extreme cold but increases humidity fluctuations and freeze–thaw cycles affecting envelope performance [112].	•Use highly insulated, airtight vegetated envelopes (e.g., polycarbonate panels) to avoid heat loss while still providing daylight. •Incorporate a moisture-buffering substrate that can absorb indoor humidity spikes.

Moisture-related side-effects are also likely to intensify under more humid future conditions. Higher outdoor absolute humidity increases indoor moisture loads via infiltration and ventilation, while plant transpiration adds to this burden, potentially exceeding moisture-management thresholds (e.g. sustained RH > 70%) and elevating condensation and mould risk [111]. Warmer and/or more humid indoor conditions can further alter indoor microbial communities and dampness-related bioaerosol constituents (including microbial fragments and pro-inflammatory agents such as endotoxins and β-glucans) [113], [114], [115], introducing a biological dimension to IEQ management with potential implications for respiratory and allergic outcomes.

Air pollutant removal may likewise improve or deteriorate under climate change. Elevated outdoor concentrations of O_3_, VOCs and PM under warmer conditions increase infiltrating pollutant loads; vegetated walls can capture a fraction (typically 10%–30% depending on species and airflow), but particle accumulation and inadequate maintenance may also promote resuspension and secondary emissions [111], [116]. During extreme heat or smoke events, behavioural adaptation, such as reduced outdoor activity and limited window opening [117], [118], may increase the relative importance of indoor greenness and green views as restorative resources, consistent with the psycho-physiological pathways discussed in Text S5 [108], [119]. Understanding these interacting pathways is essential for climate-resilient greening design and for developing monitoring and modelling tools that support proactive, data-driven decision-making.

Current air-quality monitoring (including passive and active sampling, leaf deposition measurements, and biofilter filtration efficiency) combined with advanced building-performance modelling (e.g. EnergyPlus, CFD, multi-physics, and data-driven methods), as discussed in Sections 3, 4, provides a robust evidence base for quantifying indoor greening effects under a changing climate. Field studies demonstrate how plants influence indoor temperature, humidity and pollutant concentrations in real buildings, most commonly in offices, classrooms, and residential or commercial settings [40], [51], [53], [59]. In parallel, models can isolate the contribution of plants and vertical greening systems by representing heat–moisture exchange, airflow, and pollutant transport, enabling estimation of plant performance and CO_2_ uptake under controlled boundary conditions. By calibrating plant and IEQ parameters using current monitoring datasets and subsequently driving the same models with scenario-based future boundary conditions (such as morphed weather files and extreme-event periods) [120], researchers can test whether the cooling, humidifying, and phytoremediation benefits of indoor greening will remain effective, diminish, or become increasingly critical for occupant comfort and health. Together, these monitoring and modelling approaches support a mechanistic understanding of how indoor greening can mitigate climate-driven stresses on buildings, while also identifying conditions under which unintended consequences may emerge.

Table S10 summarises current challenges and recommended strategies for improving monitoring and modelling efficiency, alongside the expected benefits of targeted methodological advances. To remain effective under shifting climate regimes, IEQ monitoring and modelling systems must evolve from static, steady-state representations toward dynamic, scenario-driven, and plant-responsive frameworks. Future approaches should integrate downscaled climate-scenario data to forecast indoor cooling performance, humidity loads, pollutant concentrations, and plant physiological responses. Real-time sensing of plant variables, such as leaf temperature, substrate moisture, sap flow, and PAR, can further enable adaptive control strategies (e.g. ventilation, irrigation, and supplementary lighting) that maintain cooling efficiency while preventing excessive humidity, mould growth and bioaerosol or allergen risk. Coupled IEQ–plant models that integrate building physics, evapotranspiration dynamics, pollutant deposition, and moisture buffering can substantially enhance predictive capability. Embedding moisture-balance and pollutant-resuspension sub-models further enables early detection of unintended outcomes, including conditions under which indoor vegetation may shift from delivering net IEQ benefits to introducing moisture- or air-quality-related risks as climate stresses intensify.

## 7 Limitations and research gaps

### 7.1 Limitations

This review has several search and evidence-synthesis limitations. Firstly, the literature search was primarily based on predefined keyword combinations and Web of Science indexing. Although citation tracking and authors’ expert knowledge were used to broaden coverage, relevant studies using different terminology or indexed in other databases may have been missed. The review was also limited to English-language, peer-reviewed studies published between January 2000 and January 2026, which may introduce language and publication-type bias. Applied evidence on indoor greening may also appear in conference papers, theses, technical reports, design guidance, or industry documents outside the review scope.

Secondly, inconsistent reporting substantially undermines the comparability of iGI monitoring studies (RQ1). Core descriptors, such as greening extent, plant characteristics, room volume, and ventilation rates, are often missing or reported inconsistently, while outdoor background conditions and basic operational details are unevenly documented. This lack of standardisation hampers normalisation and cross-study comparison and limits the level of quantitative synthesis possible in this review. As a result, formal meta-analysis or pooled effect-size estimation was not feasible, and the quantitative findings were synthesised narratively and descriptively.

Thirdly, attributing observed changes in IEQ specifically to iGI remains challenging in many monitoring studies (RQ1). Indoor thermal and air-quality conditions are strongly influenced by outdoor air exchange, indoor emission sources, occupancy patterns, and window-opening behaviour (RQ2). Suitable reference spaces or well-documented before–after designs are uncommon, reducing confidence that reported effects can be primarily ascribed to iGI rather than concurrent factors. This constrained the certainty with which this review could interpret reported field-scale iGI effects as intervention-specific outcomes.

Fourthly, the generalisability of current iGI modelling results is constrained by strong scenario dependence (RQ1). Many simulations explore a limited range of room geometries, greening extents, and ventilation regimes, with validation often relying on short-term measurements or simplified experimental setups rather than operation-representative datasets (RQ1). Consequently, predicted effect sizes can be highly configuration-dependent and difficult to extrapolate across building types, climates, and iGI configurations.

Fifthly, simplified assumptions in modelling frameworks limit the practical interpretability of predicted benefits and trade-offs (RQ1). Ventilation and HVAC operation are frequently treated as static inputs, with limited representation of dynamic control strategies or occupant behaviour. Similarly, key plant and system states, such as light availability, irrigation practices, and substrate moisture, are often simplified or weakly constrained by measurements, obscuring important interactions involving humidity, comfort, and auxiliary energy or maintenance demands. These modelling simplifications restricted the extent to which this review could compare predicted iGI performance under realistic operational conditions.

Sixthly, outdoor-to-indoor pathways are rarely examined as a continuous evidence chain (RQ2). Outdoor greening and indoor greening are commonly studied in separate strands, with few studies measuring both within the same building. As a result, vegetation-driven changes in façade boundary conditions, such as airflow, moisture, and pollutant profiles, are not consistently propagated to indoor IEQ outcomes, limiting system-level interpretation and mechanistic understanding. This limited the ability of the review to evaluate cumulative or interacting effects across outdoor, façade, ventilation, and indoor greening layers.

Finally, climate-relevant evaluation of iGI remains limited in both monitoring and modelling studies (RQ3). Most evidence is based on short-term campaigns or simplified future scenarios rather than multi-season datasets that capture realistic operation and maintenance. Climate-sensitive plant responses, including water stress, phenology, and moisture dynamics, are seldom measured and only weakly represented in models, constraining confidence in net IEQ outcomes where cooling benefits interact with humidity control and mould risk. In addition, the evidence base may be affected by positive-result publication bias, as studies reporting clear iGI benefits may be more likely to be published than studies reporting neutral, limited, or adverse outcomes.

### 7.2 Research gaps

Research gaps refer here to methodological and empirical weaknesses in the wider iGI–IEQ literature. A key gap concerns the absence of an agreed minimum set of monitoring variables for iGI–IEQ studies (RQ1; Section 3). Core descriptors of iGI systems, host-space configuration, ventilation and operation, and outdoor background conditions remain poorly harmonised. Without consistent reporting, results cannot be normalised into comparable indicators, limiting robust synthesis and making uncertainty difficult to quantify across studies.

A remaining research gap concerns the limited availability of transferable, monitoring-derived evidence on effect magnitudes (RQ1). Many datasets do not allow iGI impacts to be disentangled from ventilation changes, indoor emission sources, occupancy patterns, or behavioural variability. Reported effects are often presented without consistent uncertainty ranges or sensitivity to operating conditions, which leaves the conditions under which iGI delivers reliable IEQ improvements insufficiently defined.

In modelling studies, scaling relationships that link greening characteristics and airflow regimes to normalised IEQ outcomes remain underdeveloped (RQ1). Results are typically tied to specific room sizes, greening extents, and ventilation settings. Moreover, there is a limited establishment of generalisable links between greening density, ventilation parameters, and outcome metrics. This limits extrapolation across building types and operating regimes.

Dynamic representation of plant processes and building operation is also limited in current iGI models (RQ1; Section 4). Plant physiology, light dependence, and moisture release are seldom coupled with realistic ventilation or HVAC control strategies, resulting in model outputs that reflect fixed assumptions rather than operational variability. This limits robust assessment of trade-offs and unintended consequences.

Validated multi-scale coupling between outdoor microclimate and indoor IEQ remains insufficient (RQ3). Outdoor–indoor interactions are often implemented as one-way forcing, and rarely include feedback from building operation to the local microclimate. Seasonal and inter-annual vegetation dynamics are commonly simplified as key variables, as leaf area and moisture state are rarely monitored over time, reducing transferability across climates and exposure contexts.

Finally, propagation of climate-scenario and uncertainty into iGI performance assessments remains uncommon (RQ3; Section 6). Measurement-based calibration is seldom linked systematically to future climate weather datasets, and adaptive operational responses, such as changes in ventilation control or occupant behaviour during heat events, are rarely represented. As a result, uncertainty bounds remain weak, limiting confidence in conclusions regarding climate-resilient iGI performance.

## 8 Conclusions and future outlook

This review critically synthesised current monitoring and modelling evidence on iGI impacts on IEQ, focusing on methodological comparability, attribution, validation, and uncertainty when translating reported benefits into evidence-based decision-making.

1) Existing monitoring and modelling methods provide important insights into the effects of iGI on IEQ but inconsistent reporting, short-term studies, and limited validation reduce their effectiveness for robust decision-making.
2) Indoor and outdoor greening operate as interconnected systems influencing thermal comfort, pollutant levels, moisture, acoustics and daylight; however, integrated studies capturing such multi-scale interactions remain scarce.
3) The benefits of iGI, including pollutant removal and microclimate regulation, are highly context-dependent, shaped by ventilation, plant species, lighting, and climate, with potential trade-offs such as increased humidity and maintenance requirements.
4) Climate change is expected to intensify variability in iGI performance, enhancing evapotranspiration cooling effects in some climates, while increasing moisture-related risks and operational challenges in others.
5) To advance the field, standardized monitoring protocols, integrated and validated modelling frameworks, and climate-sensitive assessments are essential for generating transferable, evidence-based guidance for sustainable building design.

Five integrated recommendations, each linked to the relevant research question(s), should guide future iGI–IEQ research.

**Diversify iGI research and contextualise evaluation (RQ1, RQ3).** Expand research beyond living-wall dominated studies to include a range of realistic systems such as potted plants, multi-pot layouts, indoor trees, hydroponic systems, and active biofilters. Characterise outdoor contexts (weather, pollution, ventilation) and evaluate seasonal performance and stress-period performance (heatwaves, pollution episodes), to enhance real-world applicability and relevance.

**Establish measurable and modellable performance metrics with clear monitoring-simulation alignment (RQ1).** Report monitored data and model outputs under identical operating conditions (occupancy, ventilation, HVAC control, indoor activities) with precisely defined endpoints (CO_2_ dynamics, temperature/humidity, air exchange, pollutant removal, and energy or ventilation impacts where relevant). This alignment transforms case-specific findings into comparable evidence.

**Standardise performance metrics and reporting protocols (RQ1).** Develop measurable metrics that align monitoring data with modelling approaches. Consistently document core context including iGI characteristics, space geometry, ventilation, outdoor conditions, occupancy patterns and activities affecting IEQ. Normalise outcomes to improve cross-study comparison without altering study design.

**Present modelling results with transparent assumptions and validation (RQ1).** Explicitly state and trace inputs and boundary conditions, including greening descriptors, airflow regime, and operational settings. Summarise validation consistently, specifying comparison parameters and conditions. Include sensitivity and uncertainty analysis with performance claims, so that model predictions are interpreted as bounded estimates rather than fixed outcomes.

**Explicitly integrate outdoor–indoor interactions and climate sensitivity (RQ2, RQ3).** Report façade and ventilation interface conditions alongside indoor outcomes to characterise the pathway linking outdoor microclimate and pollution to indoor conditions, even without fully coupled workflows. Incorporate climate-relevant scenarios and transparently report uncertainty and boundary conditions affecting ventilation, moisture and pollution dynamics.

Across RQ1–RQ3, future iGI–IEQ research should converge around three linked priorities: evidence, methods, and transferability. The evidence base requires longer-term datasets that document iGI condition, maintenance, ventilation and occupant behaviour under representative operations. Methods need tighter monitoring–modelling integration through measurement-based calibration, explicit uncertainty reporting, and plant responses parameterised from observable inputs. Transferability requires treating outdoor conditions and climate variability as explicit drivers within the same evidence chain, ensuring findings remain applicable across building types, climates, and extreme events.

## CRediT authorship contribution statement

**Prashant Kumar:** Writing – review & editing, Writing – original draft, Visualization, Supervision, Software, Project administration, Methodology, Investigation, Funding acquisition, Formal analysis, Conceptualization. **Elisa Valentim Goulart:** Writing – review & editing, Writing – original draft, Visualization, Validation, Methodology, Formal analysis, Data curation. **Hao Sun:** Writing – review & editing, Writing – original draft, Visualization, Validation, Methodology, Formal analysis, Data curation. **John Kaiser Calautit:** Writing – review & editing, Writing – original draft, Visualization, Validation, Methodology, Data curation. **Karina Corada Pérez:** Writing – review & editing, Writing – original draft, Visualization, Methodology, Data curation. **Wenjie Song:** Writing – review & editing, Visualization, Methodology, Data curation. **Sandra Giulia Linnea Persiani:** Writing – review & editing, Methodology, Data curation. **Kamaldeep Bhui:** Writing – review & editing, Funding acquisition. **Douglas Booker:** Writing – review & editing, Investigation. **Brian Considine:** Writing – review & editing, Investigation. **Hossein Fatahian:** Writing – review & editing, Investigation. **Bruño Fraga:** Writing – review & editing, Investigation. **Bruno Furieri:** Writing – review & editing, Investigation. **Yuhan Huang:** Writing – review & editing, Investigation. **Peter J. Irga:** Writing – review & editing, Investigation. **Laurence Jones:** Writing – review & editing, Investigation. **Priyanka Kulshreshtha:** Writing – review & editing, Investigation. **Ashok K. Luhar:** Writing – review & editing, Investigation. **Giovanni Lo lacono:** Writing – review & editing, Investigation. **Maria Clara Vieira Martins Starling:** Writing – review & editing, Investigation. **Aonghus McNabola:** Writing – review & editing, Investigation. **Rakesh Mishra:** Writing – review & editing, Investigation. **Lidia Morawska:** Writing – review & editing, Investigation. **Zaheer A. Nasir:** Writing – review & editing, Investigation, Funding acquisition. **James O'Donnell:** Writing – review & editing, Investigation. **Hamid Omidvarborna:** Writing – review & editing, Investigation. **Christian Pfrang:** Writing – review & editing, Investigation. **Priyadarsini Rajagopalan:** Writing – review & editing, Investigation. **Neyval Costa Reis Junior:** Writing – review & editing, Investigation. **Robyn Schofield:** Writing – review & editing, Investigation. **Jane Meri Santos:** Writing – review & editing, Investigation. **Elson Silva Galvão:** Writing – review & editing, Investigation. **Divyanshu Sood:** Writing – review & editing, Investigation. **Taciana Toledo de Almeida Albuquerque:** Writing – review & editing, Investigation. **Jannis Wenk:** Writing – review & editing, Investigation, Funding acquisition.

## Declaration of competing interest

The authors declare that they have no known competing financial interests or personal relationships that could have appeared to influence the work reported in this paper.
