# Supplementary material for: Monitoring and modelling of indoor environmental quality to guide indoor greening applications
Source: Eco Environ Health. 2026 Oct 9;5(4):100289. doi: 10.1016/j.eehl.2026.100289 (PMC13770677; doi:10.1016/j.eehl.2026.100289)
Supplement: Supplementary file 1 [file mmc1.docx]

**Supplementary Information (SI)**

for

**Monitoring and modelling of indoor environmental quality to guide indoor greening applications**

Prashant Kumar^a,b,u,*^, Elisa Valentim Goulart^a,c^, Hao Sun^a^, John Kaiser Calautit^d^, Karina Corada Pérez^e^, Wenjie Song^d^, Sandra Giulia Linnea Persiani^f^, Kamaldeep Bhui^g^, Douglas Booker^h^, Brian Considine^i^, Hossein Fatahian^j^, Bruño Fraga^k^, Bruno Furieri^c^, Yuhan Huang^l^, Peter J. Irga^m^, Laurence Jones^n,o^, Priyanka Kulshreshtha^p^, Ashok K. Luhar^q^, Giovanni Lo laconno^r,b^, Maria Clara Vieira Martins Starling^s^, Aonghus McNabola^i^, Rakesh Mishra^t^, Lidia Morawska^u^, Zaheer A Nasir^v^, James O’Donnell^w, x^, Hamid Omidvarborna^y^, Christian Pfrang^z^, Priya Rajagopalan^aa^, Neyval Costa Reis Junior^c^, Robyn Schofield^ab^, Jane Meri Santos^c^, Elson Silva Galvão^c^, Divyanshu Sood^x^, Taciana Toledo de Almeida Albuquerque^s^, Jannis Wenk^ac,ad^

^a^Global Centre for Clean Air Research (GCARE), School of Engineering, Civil and Environmental Engineering, Faculty of Engineering and Physical Sciences, University of Surrey, Guildford GU2 7XH, United Kingdom

^b^Institute for Sustainability, University of Surrey, Guildford GU2 7XH, United Kingdom

^c^Department of Environmental Engineering, Federal University of Espírito Santo (UFES), Vitória 29075-910, ES, Brazil

^d^Department of Architecture and Built Environment, University of Nottingham, Nottingham NG7 2RD, United Kingdom

^e^Sustainability Research Institute, University of East London, London E16 2RD, United Kingdom

^f^Department of Architecture, TUM School of Engineering and Design, Technical University of Munich, Germany

^g^Department of Psychiatry and Wadham College, University of Oxford; Oxford Health NIHR BRC; Public Mental Health Implementation Centre at Royal College of Psychiatrists, Oxford, OX3 7JX, United Kingdom

^h^School of Civil Engineering, University of Leeds, Leeds, LS2 9LG, United Kingdom

^i^School of Engineering, RMIT University, Melbourne, Australia

^j^School of Computing and Engineering, University of Huddersfield, Huddersfield HD1 3DH, United Kingdom

^k^School of Engineering, University of Birmingham, Edgbaston, B15 2TT, United Kingdom

^l^Centre for Green Technology, School of Civil and Environmental Engineering, University of Technology Sydney, NSW 2007, Australia

^m^Plants and Environmental Quality Research Group, School of Civil and Environmental Engineering, University of Technology Sydney, NSW 2007, Australia

^n^UK Centre for Ecology & Hydrology, Environment Centre Wales, Bangor LL57 2UW, United Kingdom

^o^Liverpool Hope University, Department of Geography and Environmental Science, Hope Park, Liverpool L16 9JD, United Kingdom

^p^Bhagini Nivedita College, University of Delhi; Society for Indoor Environment, India

^q^CSIRO Environment, Aspendale, Victoria, Australia

^r^School of Veterinary Medicine, Faculty of Health and Medical Sciences, University of Surrey, Guildford, GU2 7AL, United Kingdom

^s^Department of Sanitary and Environmental Engineering, School of Engineering, Federal University of Minas Gerais, Belo Horizonte 31270-901, Brazil

^t^School of Computing and Engineering, University of Huddersfield, Huddersfield, HD1 3DH, United Kingdom

^u^Queensland University of Technology, International Laboratory for Air Quality and Health, Brisbane, QLD 4000, Australia

^v^Faculty of Engineering and Applied Sciences, Cranfield University, Cranfield MK43 0AL, United Kingdom

^w^School of Engineering, University of Limerick, Limerick, Ireland

^x^School of Mechanical and Materials Engineering; UCD Energy Institute, Dublin, Ireland

^y^Mobile Source Laboratory Division, California Air Resources Board, Riverside, CA 92507, USA

^z^School of Geography, Earth and Environmental Sciences, University of Birmingham, Edgbaston, Birmingham, B15 2TT, United Kingdom

^aa^School of Property, Construction and Project Management, RMIT University, Melbourne, Australia

^ab^School of Geography, Earth and Atmospheric Sciences, University of Melbourne, Parkville, Australia

^ac^Division G - Qualitative Hydrology, Department G1 - General Water Quality Issues, Federal Institute of Hydrology (BfG), Am Mainzer Tor 1, 56068 Koblenz, Germany

^ad^Department of Chemical Engineering, University of Bath, Bath, BA2 7AY, United Kingdom

*Corresponding author.

Email: [p.kumar@surrey.ac.uk](mailto:p.kumar@surrey.ac.uk) (P. Kumar)

**This document includes:**

- Section S1-S7
- Figures S1-S3
- Tables S1-S10

**S1.	Cluster analyses**

A bibliometric analysis was performed using VOSviewer software version 1.6.1948 for quantitative and qualitative analysis to identify trends from the selected papers. Keywords were extracted via VOSviewer and a matrix created based on their frequency, co-occurrence and similarity. Keywords with high similarity and co-occurrence were grouped closely together, while less similar ones were spaced further apart.

To characterise dominant research themes, a keyword co-occurrence network of 207 keywords (each appearing at least four times) was created and organised into four clusters (Figure S2). Cluster #1 (17 items) centres on “air pollution indoor” and includes themes such as “air pollutants/analysis”, “volatile organic compounds”, “environmental monitoring”, and “humans”, reflecting a strong focus on indoor air pollution monitoring and human exposure. Cluster #2 (6 items) groups terms related to “indoor pollutants”, such as “carbon dioxide”, “formaldehyde”, “indoor air quality”, “indoor plants”, and “volatile organic compounds”, highlighting research on indoor pollutant dynamics and the role of plants in influencing indoor air quality particularly regarding gaseous pollutants. Cluster #3 (4 items) represents a smaller, more specialised theme focused on modelling and simulation approaches; keywords such as “EnergyPlus”, “machine learning”, “evapotranspiration” and “living walls” point to an emerging computational research direction. Cluster #4 (3 items) centres on “phytoremediation” and includes terms such as “green walls” and “living walls”. Overall, the bibliometric map indicates that IEQ research primarily focuses on indoor air pollution characterisation and human exposure, with growing interest in iGI interventions, such as indoor plants, living walls and green walls, as potential mitigation strategies. It also reveals a clear methodological imbalance, with experimental and monitoring-based studies dominating, while modelling approaches remain comparatively underrepresented but are beginning to emerge.

**S2.	Lighting effect on CO_2_ removal and noise reduction from monitored green wall**

Green walls often exhibit limited CO₂ removal under real indoor conditions, primarily because in-situ lighting is typically inadequate to sustain effective photosynthesis. Chamber experiments demonstrate that CO₂ uptake can be substantially enhanced under optimised lighting, particularly when light intensity (up to 200 µmol m⁻² s⁻¹), incidence angle (15–45°), and plant phototropism are carefully controlled, underscoring the need for clear technical lighting design guidelines for indoor living walls [1]. Tested species (*Peperomia obtusifolia*, *Tradescantia spathacea*, *Chlorophytum comosum*, *Spathiphyllum wallisii*, *Aeschynanthus radicans*, and *Philodendron hederaceum)* show tolerance to low-light environments, but their CO₂ removal potential remains limited without lighting optimisation [2]. Beyond air quality, green walls delivered clear acoustic benefits, increasing sound insulation by ~6 dB (up to 10 dB at 800 Hz) and reducing reverberation across low and high frequencies, thereby improving overall indoor acoustic comfort [3].

**S3. Passive green wall for CO_2_ reduction**

Passive indoor green walls are often assumed to improve IAQ, but indoor light levels determine whether plants act as net CO₂ sinks or sources. Jafar et al. [4] address this uncertainty with a parametric digital workflow linking daylight simulation to plant physiological response to estimate CO₂ performance at early design stage. The case models a hypothetical single-zone room (192 m³) containing a 3 m² living wall planted with Epipremnum aureum, selected for shade tolerance and well-characterised CO₂-light response. Radiance-based daylight simulation maps spatially-resolved photosynthetically active radiation (PAR) onto leaf elements converting it into CO₂ uptake or release using empirical photosynthesis and respiration. Instead of modelling time-dependent CO₂ concentrations, the approach evaluates the overall balance to test the effect of wall location, orientation,window-to-wall ratio, and indoor light regime. The results demonstrate a clear threshold: under sufficiently illuminated conditions (PAR above roughly 70 μmol m⁻² s⁻¹), the living wall predicts a reduction of indoor CO₂ by ~ 3–3.6 ppm h^–1^, corresponding to uptake rates up to ~1600 mg CO₂/h; under low-light conditions (PAR below roughly 25 μmol m⁻² s⁻¹), the same system becomes a net emitter, increasing indoor CO₂ by up to around 0.8 ppm h^–1^. This case study corroborates the condition-dependent nature of passive green walls as IEQ interventions, whose CO₂ benefits are not guaranteed. More modelling efforts covering a wider parameter space and supporting datasets are critical to improve our understanding of this biotechnology and assess the benefits of interventions.

**S4.	Active green walls for ventilation enhancement**

Passive green-wall modelling rarely translates “greening benefits” into explicit ventilation performance gains, because airflow is often prescribed through fixed ACH assumptions rather than treated as an outcome in its own right. An active green-wall case addresses this limitation by configuring iGI as a mechanically assisted airflow device and evaluating its impact using room-scale airflow modelling. Kanbur et al. [5] examine an L-shaped office with a floor area of ~160 m² and a volume of ~480 m³, coupled to a fan-driven plant-based green wall comprising 128 plants mounted on a porous planting layer. By treating the green wall as a flow-through element, the model directly quantifies ventilation renewal rather than inferring it indirectly from pollutant decay. The simulations indicate substantial increases in ACH, with predicted improvements ranging from roughly 38%–95% depending on zone location, and the largest gain occurring in the region closest to the green wall. Importantly, these ventilation enhancements are not accompanied by widespread high-velocity discomfort in the occupied zone: most indoor air speeds remain within approximately 0.01–0.3 m s^-1^, with only localised peaks around 0.5 m s^-1^ near geometric discontinuities. This case therefore demonstrates that, when iGI is designed as an active system with forced airflow, it can function as a ventilation-performance intervention that measurably increases ACH and redistributes air within a room, while largely maintaining air speeds within commonly acceptable indoor ranges [5].

**S5. Partly transitional green walls for CO_2_ control**

Indoor green walls are sometimes treated as inherently beneficial for CO₂ control, yet their net effect is tightly constrained by both ventilation capacity and how ventilation is operated in practice. Mileikovskyi et al. [6] illustrate this point under natural ventilation conditions by coupling a phytowall CO₂ source and sink representation with an analytical ventilation model designed for residential settings where low wind speeds and small temperature differences are common. Using Aglaonema roebelinii physiology, a whole-wall phytowall can act as a daytime CO₂ sink, with an uptake rate of ~1.56 × 10⁻⁴ kg s^–1^, but that the benefit can reverse at night when respiration produces a comparable CO₂ release of about 1.63×10⁻⁴ kg s^–1^ [6]. The analysis further shows that when wind speeds are below roughly 0.25 m s^–1^ and buoyancy driving is weak, natural ventilation alone may be insufficient to prevent indoor CO₂ from exceeding 1000 ppm, even with the green wall present, underscoring that plant exchange cannot compensate for inadequate air renewal. A complementary operational perspective [7] links living-wall CO₂ removal to ventilation control behaviour in an office setting at minute resolution. Rather than assuming a fixed ACH, their case demonstrates how plant uptake can delay or reduce ventilation demand, for example delaying the onset of ventilation by ~17 minutes for two occupants and reducing required outdoor airflow under variable air volume operation, but only within the limits imposed by the selected CO₂ thresholds and control logic [7]. Together, these cases position partly transitional green walls as CO₂-relevant interventions whose performance depends less on the presence of vegetation alone and more on the coupled system of plant metabolism, ventilation driving forces, and HVAC control strategy.

**S6.	Active biofilters for rapid pollutant removal**

Biofilters represent a device-like branch of iGI in which vegetation is positioned primarily as a functional removal medium, shifting the modelling focus from envelope-scale hygrothermal effects to short-term pollutant decay and the airflow penalties that determine deployability. Two Azolla-based studies illustrate this rapid-removal framing while highlighting that performance claims are inseparable from pressure-drop and flow constraints. Vivekanandan et al. [8] use CFD to assess an Azolla biofilter concept in a room-scale setting, modelling pollutant reduction as a fast process and reporting that, within a short window of ~300 s, CO₂ can decrease by ~40% and PM₂.₅ by ~55%, suggesting strong potential for rapid concentration suppression under the assumed operating conditions [8]. Moving from “how fast can it clean?” to “can it be integrated into ventilation?”, Vivekanandan and Rajeswari [9] combine a well-mixed ODE decay model with CFD-predicted airflow and porous-media representations to evaluate an outlet-mounted Azolla filter panel. In a 36 m³ test room at ~2 ACH, a 0.25 m² biofilter achieves high short-term particle removal, rising from ~29% at 5 minutes to ~85% at 30 minutes, with size-resolved efficiencies after 30 minutes ranging from roughly 77% (1 μm) to 95% (10 μm) for microplastic particles [9]. Crucially, the same case explicitly reports the aerodynamic cost, with a pressure drop of 0.14 Pa for the 0.25 m² panel, and explores how panel area influences ΔP, making it clear that filter effectiveness must be evaluated alongside resistance and face-velocity considerations. Together, these studies demonstrate the promise of plant-based biofilters for rapid pollutant removal, but also show that their practical value depends on jointly meeting removal targets and ventilation-design constraints rather than on removal efficiency alone.

**S7.	Psycho-physiological and behavioural pathways of indoor-outdoor greenery**

A parallel body of research examines indoor and outdoor greenery as joint exposures influencing mental health and behaviour, often independently of (or without directly measuring) physical IEQ. During COVID-19 lockdowns, Zhang et al. [10] showed in Shanghai that both indoor greenery (houseplants, gardening, digital nature, green views) and outdoor greenery (NDVI within 300 m and perceived community greenness) were linked to lower depressive symptoms: structural equation modelling suggested greener homes/neighbourhoods increased perceived restorativeness, reducing loneliness and fear of COVID-19. National surveys in Italy and Bulgaria [11,12] similarly found indoor plants, sunlight, green views, private green space and neighbourhood greenness buffered increases in anxiety, sleep disturbance and depression; Paniccià et al. [13] synthesise broader evidence for restorative and stress-buffering pathways. However, these studies rarely measure physical IEQ (greenness is typically NDVI/self-report/green-view indices), underscoring the need for frameworks that link measured IEQ mediation with psycho-physiological outcomes.

**Figure S1.** PRISMA flowchart diagram showing identification, screening, eligibility, and inclusion. Counts are based on combined keyword results summarised in Table S3. The final evidence base is dominated by monitoring-based studies (Topic A), with fewer modelling-based contributions (Topic B) and notable research gaps in indoor–outdoor interactions (Topic C) and climate-oriented perspectives (Topic D). This distribution highlights the fragmented nature of the current indoor greening literature.

**Figure S2.** Keyword co-occurrence network. Frequently occurring keywords (≥4 occurrences) in the reviewed studied were grouped into four clusters: Cluster #1 (with main keyword “Air pollution indoor”), Cluster #2 (“Indoor pollutants”), Cluster #3 (“Computational approaches”), and Cluster #4 (“Phytoremediation”).

**Figure. S3**. Climate-change pathways linking outdoor drivers to façade and ventilation boundary conditions, indoor loads, and the balance between iGI and IEQ benefits and moisture- or air-quality-related IEQ risks.

**Table S1**. Summary of previous reviews on various aspects of indoor greening and IEQ.

| **iGI type**  **(Location)** | **Focus area** | **Key findings** | **Reference** |
| --- | --- | --- | --- |
| 26 iGI types (residential & commercial) | Multi-dimensional iGI–IEQ interactions (air quality, thermal comfort, humidity, microbiome, health, equity, implementation). | - Synthesises evidence across 10 questions and 5 thematic clusters; introduces a matrix linking iGI types to 20 IEQ parameters. iGI can improve air quality, regulate humidity, and enhance thermal comfort; effectiveness depends on species, density, and ventilation integration. Potential benefits include enriched indoor microbial ecosystems and provide psychological/cognitive improvements when moisture/airflow are well managed. - Major data gaps persist (long-term field evidence, microbial dynamics, socio-economic accessibility); widespread adoption requires overcoming technical and regulatory barriers. | Kumar et al. [14] |
| Indoor potted plants / botanical biofilters | Monitoring-effects on IAQ and health; pollutant-removal performance across species. | - Indoor plants reduce VOCs, CO₂ and PM; efficiency varies by species. - Certain species (e.g., *Chlorophytum*, *Spathiphyllum*) show consistently higher removal. - Plants support wellbeing via stress reduction and improved perception. | Rasheed & Jayasree [15] |
| Indoor vertical greening system (VGS) | Monitoring environmental and health/wellbeing impacts. | - VGS improve air quality, thermal comfort and noise reduction, with strong evidence for PM removal and cooling. - Psychological benefits reported but mostly from short-term self-reports. - Objective health measurements are rare. | Fonseca et al. [16] |
| Active botanical filters | Plant-based biofiltration technologies for indoor air purification; pollutant-removal mechanisms. | - Active filters can remove >50% of VOCs/PM; performance depends on plant species and system design. - Root microbe interactions play a major role in VOC degradation. | Montaluisa-Mantilla et al. [17] |
| Indoor potted plants and passive indoor greening systems | Monitoring effects on thermal conditions, IAQ (VOCs & CO₂), and wellbeing;  impacts on learning/productivity, emotional state, and patient recovery. | - Plants lower VOCs and CO₂ and improve thermal comfort via transpiration. - Psychological benefits include improved attention, reduced fatigue, and faster patient recovery. - Performance varies widely by species; quantitative real-world evidence remains limited. | Liu et al. [18] |
| Indoor potted plants (experimental and real-room environments) | Quantitative monitoring of pollutant removal (formaldehyde, benzene, toluene) and microclimate impacts | - Plants reduce key pollutants and can increase humidity and lower temperature. - Effects vary by species; mixed setups improve outcomes. - Most evidence is lab-based; real-world validation and standardised protocols are lacking. | Han & Ruan [19] |
| Indoor living walls / vertical greening (commercial & residential) | Monitoring and modelling of microclimate, IAQ, energy, wellbeing, and risks. | - Most evidence from exterior studies; indoor benefits uncertain. - Possible advantages include modest cooling, IAQ improvement, acoustic benefits and wellbeing. - Risks (VOCs, CO₂ respiration, humidity, allergens) require targeted indoor studies. | Gunawardena & Steemers [20] |
| Indoor potted plants | Physiological, psychological, and air-purification effects; mechanisms of photosynthesis, transpiration, and pollutant removal. | - Plants contribute to CO₂ reduction, humidity regulation, and VOC removal. - Psychological benefits include reduced stress and improved task performance. - Plants offer multi-functional potential for air purification and comfort enhancement. | Deng & Deng [21] |
| Indoor green systems (potted plants, living walls, biofilters) | Effects on IEQ (air quality, thermal, acoustic, psychological); pollutant removal pathways and, plant–microbe interactions. | - Indoor greenery improves IAQ via VOC removal through leaves, roots, and microbes. - Additional benefits include cooling via evapotranspiration, noise reduction, and psychological well-being. - True pollutant-removal performance in real environments remains unclear; active systems show higher potential than passive. | Armijos Moya et al. [22] |
| Ornamental potted plants | Capacity to remove VOCs from indoor air. | - Ornamental plants can reduce certain VOCs (e.g., formaldehyde, benzene, toluene), but removal magnitude is modest and depends on plant selection, density, and ventilation. | Dela Cruz et al. [23] |

**Table S2**. Targeted keyword groups were used to search the literature published between January 2000 and December 2025, detailing the specific terms applied within each group and the corresponding number of records initially retrieved.

| **Category** | **Search terms** | **Paper quantity** |
| --- | --- | --- |
| **Indoor greening types^a^** | "Indoor greening" OR "indoor green infrastructure" OR "indoor vegetation" OR "green wall" OR "living wall" OR "vertical greenery system" OR "potted plants" OR "planter box" OR "desktop plants" OR "indoor trees" OR "moss wall" OR "biofilter wall" OR "hydroponic system" OR "aquaponic system" OR "interior landscaping" OR "biophilic design" | 6,421 |
| **Monitoring approaches^b^** | "Environmental monitoring" OR "indoor environmental monitoring" OR "sensor network" OR "low-cost sensors" OR "IoT monitoring" OR "data acquisition" OR "wireless sensor network" OR "real-time monitoring" OR "microclimate monitoring" OR "indoor air quality monitoring" OR "temperature and humidity sensors" OR "CO₂ monitoring" OR "particulate matter monitoring" OR “leaf sampling” or “bacterial sampling” OR “watering quantity” | 224,777 |
| **Modelling approaches^c^** | “computational fluid dynamics" OR "CFD simulation" OR "numerical modelling" OR "energy simulation" OR "thermal modelling" OR "indoor airflow simulation" OR "heat transfer model" OR "mass balance model" OR "air quality modelling" OR "plant transpiration model" OR "photosynthesis model" OR "machine learning" OR "data-driven modelling" OR "hybrid modelling" OR “ANSYS Fluent” OR “OpenFOAM” OR "ENVImet" OR “indoor air modelling” OR “dispersion modelling” OR “mass balance model” OR “multi-zone model” OR “simulation of VOC removal” | 733,224 |
| **IEQ parameters^d^** | ("indoor air quality" OR "CO₂ concentration" OR “O2” OR “O3” OR "PM1" OR "PM2.5" OR "PM10" OR “particulate matter” OR "VOC" OR "formaldehyde" OR "temperature" OR "humidity" OR “relative humidity” OR "thermal comfort" OR "air velocity" OR "daylighting" OR "illuminance" OR "sound level" OR "noise reduction" OR "indoor climate" OR "microclimate" OR “noise” OR “acoustic”) | 5,193,066 |
| **Indoor outdoor interaction^e^** | "Office building" OR "residential building" OR "school classroom" OR "hospital ward" OR "indoor workspace" OR "urban apartment" OR "green retrofitting" OR "low-carbon building" OR "zero-carbon building" OR "building envelope greening" | 19,757 |
| **Changing climate^f^** | "Energy efficiency" OR "passive cooling" OR "passive design" OR "indoor carbon sequestration" OR "sustainable building design" OR "nature-based solution" OR "climate adaptation" OR "net-zero building" OR "resilient building design" | 183,293 |

Note: Combination of keywords used to identify publications and extract data for Section 3^a,b,d,^ Section 4^a,c,d^, Section 5^a,d,e^, and Section 6^a,d,f^.*.*

**Table S3.** Number of studies identified, screened, and retained for data extraction based on combined keyword groups in literature searches published between January 2000 and December 2025.

| Combined key words | Paper identified through database searching | Papers screened based on title, keywords and abstract | Paper includes for data extraction and analyses |
| --- | --- | --- | --- |
| Topic A   - Indoor greening types - IEQ parameters - Monitoring approaches | 54 | 50 | 48 |
| Topic B   - Indoor greening types - IEQ parameters - Modelling approaches | 37 | 15 | 11 |
| Topic C   - Indoor greening types - IEQ parameters - Indoor outdoor interaction | 12 | 7 | 1 |
| Topic D   - Indoor greening types - IEQ parameters - Changing climate | 89 | 0 | 0 |

**Table S4.** Inventory and frequency of indoor plant species reported across the reviewed studies, classified by functional plant type and common English name.

| **Plant name** | **Quantity** | **Type** | **Common name (English)** |
| --- | --- | --- | --- |
| *Adiantum sp.* | 3 | Ferns | Maidenhair fern |
| *Aeschynanthus radicans* | 3 | Climbing species / vines | Lipstick plant |
| *Aglaonema commutatum* | 3 | Herbaceous | Chinese evergreen |
| *Aglaonema modestum* | 1 | Herbaceous | Chinese evergreen |
| *Anthurium andraeanum* | 1 | Herbaceous | Flamingo flower |
| *Aptenia cordifolia* | 1 | Herbaceous | Baby sun rose |
| *Arachniodes aristata* | 1 | Ferns | East Asian holly fern |
| *Araucaria heterophylla* | 1 | Trees | Norfolk Island pine |
| *Ardisia crenata* | 1 | Shrubs | Coralberry |
| *Ardisia pusilla* | 1 | Herbaceous | Dwarf ardisia |
| *Asparagus sprengeri* | 1 | Herbaceous | Asparagus fern |
| *Asplenium antiquum* | 1 | Ferns | Japanese bird’s nest fern |
| *Asplenium nidus* | 2 | Ferns | Bird’s nest fern |
| *Botrychium ternatum* | 1 | Ferns | Moonwort fern |
| *Calathea spp.* | 3 | Herbaceous | Prayer plant |
| *Camellia japonica* | 1 | Shrubs | Japanese camellia |
| *Camellia sinensis* | 1 | Shrubs | Tea plant |
| *Carpobrotus edulis* | 1 | Trees | Ice plant |
| *Chamaecyparis obtusa* | 1 | Trees | Hinoki cypress |
| *Chamaedorea elegans* | 2 | Palms | Parlor palm |
| *Chlorophytum bichetii* | 1 | Herbaceous | Spider plant |
| *Chlorophytum comosum* | 16 | Herbaceous | Spider plant |
| *Chlorophytum orchidastrum* | 1 | Herbaceous | Fire flash plant |
| *Chrysalidocarpus lutescens* | 2 | Palms | Areca palm |
| *Chrysanthemum morifolium* | 1 | Herbaceous | Florist’s chrysanthemum |
| *Cissus rhombifolia* | 1 | Climbing species / vines | Grape ivy |
| *Clivia miniata* | 1 | Herbaceous | Kaffir lily |
| *Coniogramme japonica* | 1 | Ferns | Bamboo fern |
| *Cupressus macrocarpa* | 1 | Trees | Monterey cypress |
| *Cycas revoluta* | 1 | Trees | Sago palm |
| *Cyrtomium caryotideum* | 1 | Ferns | Holly fern |
| *Cyrtomium falcatum* | 1 | Ferns | Japanese holly fern |
| *Davallia fejeensis* | 2 | Ferns | Rabbit’s foot fern |
| *Davallia mariesii* | 1 | Ferns | Squirrel’s foot fern |
| *Dendropanax morbifera* | 1 | Trees | Korean dendropanax |
| *Dieffenbachia amoena* | 1 | Herbaceous | Dumb cane |
| *Dieffenbachia compacta* | 1 | Herbaceous | Dumb cane |
| *Dieffenbachia maculata* | 1 | Herbaceous | Dumb cane |
| *Dizygotheca elegantissima* | 1 | Herbaceous | False aralia |
| *Dracaena* | 1 | Trees | Dragon tree |
| *Dracaena concinna* | 1 | Trees | Dragon tree |
| *Dracaena deremensis* | 4 | Trees | Dracaena |
| *Dracaena fragrans* | 3 | Trees | Corn plant |
| *Dryopteris erythrosora* | 2 | Ferns | Autumn fern |
| *Dryopteris nipponensis* | 1 | Ferns | Japanese wood fern |
| *Elaeocarpus sylvestris* | 1 | Trees | Evergreen elaeocarpus |
| *Epipremnum aureum* | 11 | Climbing species / vines | Golden pothos |
| *Eugenia myrtifolia* | 1 | Shrubs | Australian brush cherry |
| *Eurya emarginata* | 1 | Shrubs | Eurya |
| *Fatsia japonica* | 2 | Shrubs | Japanese aralia |
| *Ficus benjamina* | 4 | Trees | Weeping fig |
| *Ficus elastica* | 2 | Trees | Rubber plant |
| *Ficus lyrata* | 1 | Trees | Fiddle-leaf fig |
| *Ficus pumila* | 5 | Climbing species / vines | Creeping fig |
| *Gardenia jasminoides* | 1 | Herbaceous | Cape jasmine |
| *Gibasis sp.* | 1 | Climbing species / vines | Tahitian bridal veil |
| *Haemaria discolor* | 1 | Herbaceous | Jewel orchid |
| *Hedera helix* | 4 | Climbing species / vines | English ivy |
| *Howea belmoreana* | 1 | Palms | Sentry palm |
| *Hoya carnosa* | 1 | Shrubs | Wax plant |
| *Hoya pubicalyx ‘Royal Hawaiian’* | 1 | Shrubs | Wax plant |
| *Ilex crenata* | 1 | Shrubs | Japanese holly |
| *Jasminum polyanthum* | 1 | Shrubs | Pink jasmine |
| *Jasminum sambac* | 1 | Shrubs | Arabian jasmine |
| *Kalanchoe blossfeldiana* | 1 | Shrubs | Flaming Katy |
| *Laurus nobilis* | 1 | Trees | Bay laurel |
| *Lavandula spp.* | 1 | Shrubs | Lavender |
| *Ligustrum japonicum* | 1 | Shrubs | Japanese privet |
| *Maranta leuconeura* | 2 | Herbaceous | Prayer plant |
| *Mentha suaveolens* | 1 | Herbaceous | Apple mint |
| *Microlepia strigosa* | 1 | Ferns | Lace fern |
| *Monstera deliciosa* | 2 | Climbing species / vines | Swiss cheese plant |
| *Nandina domestica* | 1 | Shrubs | Heavenly bamboo |
| *Nematanthus glabra* | 3 | Herbaceous | Goldfish plant |
| *Neomarica gracilis* | 2 | Herbaceous | Walking iris |
| *Nephrolepis biserrata* | 1 | Ferns | Giant sword fern |
| *Nephrolepis cordifolia* | 3 | Ferns | Tuberous sword fern |
| *Nephrolepis exaltata* | 6 | Ferns | Boston fern |
| *Osmunda japonica* | 1 | Ferns | Japanese royal fern |
| *Pachira aquatica* | 2 | Trees | Money tree |
| *Pelargonium spp.* | 1 | Ferns* | Geranium (*commonly herbaceous) |
| *Peperomia clusiifolia* | 1 | Herbaceous | Red-edge peperomia |
| *Peperomia magnoliifolia* | 1 | Herbaceous | Baby rubber plant |
| *Peperomia obtusifolia* | 4 | Herbaceous | Baby rubber plant |
| *Philodendron hederaceum* | 15 | Climbing species / vines | Heartleaf philodendron |
| *Philodendron selloum* | 1 | Climbing species / vines | Tree philodendron |
| *Philodendron xanadu* | 1 | Climbing species / vines | Xanadu philodendron |
| *Phoenix roebelenii* | 1 | Palms | Pygmy date palm |
| *Pittosporum tobira* | 1 | Shrubs | Japanese mock orange |
| *Plantago asiatica L.* | 1 | Climbing species / vines | Asian plantain |
| *Polypodium formosanum* | 1 | Ferns | Formosa polypody |
| *Polyscias balfouriana* | 1 | Trees | Balfour aralia |
| *Polystichum tripteron* | 1 | Ferns | Japanese shield fern |
| *Psidium guajava* | 1 | Trees | Guava |
| *Pteris dispar* | 1 | Ferns | Brake fern |
| *Pteris ensiformis* | 1 | Ferns | Sword brake fern |
| *Pteris multifida* | 1 | Ferns | Spider brake |
| *Quercus acuta* | 1 | Trees | Japanese evergreen oak |
| *Quercus glauca* | 1 | Trees | Ring-cupped oak |
| *Raphiolepis umbellata* | 1 | Shrubs | Yeddo hawthorn |
| *Rhapis excelsa* | 2 | Palms | Lady palm |
| *Rosmarinus officinalis* | 1 | Shrubs | Rosemary |
| *Saintpaulia ionantha* | 1 | Herbaceous | African violet |
| *Sansevieria trifasciata* | 4 | Herbaceous | Snake plant |
| *Schefflera arboricola* | 6 | Climbing species / vines | Dwarf umbrella tree |
| *Schefflera octophylla* | 1 | Climbing species / vines | Ivy tree |
| *Schlumbergera truncata* | 2 | Climbing species / vines | Thanksgiving cactus |
| *Selaginella tamariscina* | 1 | Ferns | Spikemoss |
| *Serissa foetida* | 1 | Shrubs | Snow rose |
| *Soleirolia soleirolii* | 1 | Herbaceous | Baby’s tears |
| *Spathiphyllum blandum* | 1 | Herbaceous | Peace lily |
| *Spathiphyllum wallisii* | 11 | Herbaceous | Peace lily |
| *Platycerium spp.* | 1 | Ferns | Staghorn fern |
| *Stauntonia hexaphylla* | 1 | Climbing species / vines | Chocolate vine |
| *Syngonium podophyllum* | 4 | Climbing species / vines | Arrowhead plant |
| *Taraxacum mongolicum* | 1 | Herbaceous | Asian dandelion |
| *Thelypteris acuminata* | 1 | Ferns | Marsh fern |
| *Thelypteris decursivepinnata* | 1 | Ferns | Japanese beech fern |
| *Thelypteris esquirolii* | 1 | Ferns | Chinese beech fern |
| *Thelypteris torresiana* | 1 | Ferns | Giant maiden fern |
| *Tillandsia cyanea* | 1 | Climbing species / vines | Pink quill |
| *Trachelospermum asiaticum* | 1 | Climbing species / vines | Asiatic jasmine |
| *Tradescantia spathacea* | 3 | Climbing species / vines | Oyster plant |
| *Tradescantia zebrina* | 2 | Climbing species / vines | Inch plant |
| *Viburnum awabuki* | 1 | Shrubs | Sweet viburnum |
| *Zamia pumila* | 1 | Trees | Coontie |
| *Zamioculcas zamiifolia* | 1 | Climbing species / vines | ZZ plant |

**Table S5.** Summary of experimental environment characteristics, room dimensions, ventilation conditions, monitoring duration, and greening scales across the reviewed iGI studies.

| Reference | Space Type | Floor Area (m2) | Room Volume (m3) | Ventilation Condition | Monitoring Duration | Greening Scale / IGI Area (m2) |
| --- | --- | --- | --- | --- | --- | --- |
| Alsadun et al. [24] | Office | 14.0 | 37.8 | Mechanically vent. (VRF) | 12 months | 5.76 |
| Yungstein & Helman [2] | Lab / Office | ~46.0 | ~140.0 | HVAC (6 ACH or 0.1 ACH) | ~15 months | 15.0 |
| Shao et al. [25] | Office Corridor | — | 30.6 | Naturally ventilated | 10 months | 10.6 |
| Scamoni et al. [3]; Salamone et al. [26] | ZEB Laboratory | 23.7 – 24.0 | 70.7 | Mechanically vent. (HR units) | 2 years | 7.0 – 7.6 |
| Shen et al. [27] | Office / Atrium | 75.0 (Office) | 146.4 (Atrium) | HVAC / Free-running | 90 days | 132.6 |
| Tudiwer & Korjenic [28] | Classroom | 54.6 | 202.0 | Naturally ventilated | 10 months | 5.5 |
| Caron et al. [29] | IRINA Exp. Room | 12.5 | 40.0 | HVAC (0.3 ACH) | Transient events | 2.7 |
| Wood et al. [30] | Staff Offices | 10.0 – 12.0 | 30.0 – 50.0 | HVAC / Naturally ventilated | 6 months | 3 or 6 potted plants |
| Shao et al. [31] | Hall / Office | — | 88.92 | Unknown | — | 6.86 |
| Su & Lin [32] | Laboratory | — | 38.88 | Unknown | — | 5.72 |
| Poorova & Vranayova [33] | Classroom | — | 207.2 | Unknown | — | 3.0 |
| Iddio et al. [34] / Wyoming | Conference Room | 30.25 | 90.75 | HVAC | — | 25% LFAR |
| Ding et al. [35] | Lab rooms | — | 25.2 | Mechanically vent. (Split AC) | 5 days | 140 plants |
| Shen et al. [36] | Large Chambers | — | ~3.0 | Sealed chamber (1.5 ACH) | 12 days | 1 trimmed potted plant |
| Abedi et al. [37] | Sealed Chamber | — | 0.24 (240 L) | Sealed chamber | 6 hours | Portable biofilter |
| Teiri et al. [38] | Sealed Chamber | — | 0.375 (375 L) | Sealed chamber | ~2 weeks | 2 potted plants |
| Ghazalli et al. [39] | Office Corridor | 1.8m wide / 37m long | — | Naturally ventilated | 4 months | ~3.25 |
| Irga et al. [40] | Glass Chambers | — | 15 L / ~43.0 | Sealed chamber | ~24-48 hours | 1 plant / 1.0 |
| Dominici et al. [1] | Comm. Buildings | — | Variable | HVAC | 10-day / In-situ | 16.25 – 60.0 |
| Lin et al. [41] | Modeling Box | — | 0.225 | Sealed chamber | Multi-day cycles | 1 potted plant |
| Hörmann et al. [42] | Biobox / Chamber | — | 0.24 (240 L) | Sealed chamber | 48 hours | Potted plants |
| Pettit et al. [43] | Sealed Chamber | — | 0.216 (216 L) | Sealed chamber | Short-term | 0.25 module |
| Dela Cruz et al. [44] | Glass Chambers | — | 0.0575 (57.5 L) | Sealed chamber | ~24-48 hours | 1 potted plant |
| Kazemi et al. [45] | Laboratory | — | Unknown | Unknown | Monthly samples | Modular structure |

**Table S6**. Summary of reported indoor thermal and humidity effects associated with iGI.

| **Reference** | **iGI Type** | **Temperature** | **RH** | **Potential Negative Effect** |
| --- | --- | --- | --- | --- |
| Yang et al. [46] | Green wall | ↓0.6 °C | ↑4.3-5% |  |
| Yungstein & Helman [2] | Green wall | ↓2.5–4.5°C  HVAC 1.2–3.6°C | ↑18.2% | **-** |
| Ghazalli et al. [39] | Green wall | ↓0.5–1.5 °C | ↑2–5% | **-** |
| Pérez‑Urrestarazu et al. [47] | Green wall | ↓0.8 - 4.8 ºC | - | **-** |
| Gunawardena & Steemers [48] | Green wall | ↓3% summer,  ↓6% winter | - | - |
| Qadir et al. [49] | Green wall | ↓0–4 °C | ↑7.2%-6% | Potential for respiratory discomfort in already humid climates |
| Shao et al. [25] | Green wall |  | ↑3.1–6.4%. |  |
| Salamone et al. [26] | Green wall | - | - | Oversized design causes excessive relative humidity (up to 80%) leading to thermal discomfort. |
| Abedi et al. [37] | Biofilter | ↓ 4.2 °C | ↑34.9% | Cooling efficiency declines as substrate dehydrates; higher fan power required for wet media. |
| Abdo & Huynh [50] | Biofilter | ↓ 1-1,5 °C day  ↓ 2.5–3 °C night | ↑25% | Risk of saturation (100% RH) in unventilated spaces; air backflow in certain module designs. |
| Ding et al. [35] | Biofilter | - | ↑59.6% | Increased risk of mosquito attraction and bacterial growth; slightly higher AC energy use (3.29%). |

**Table S7**. Summary of CO_2_, VOC and TVOC removal associated with iGI. Reported removal efficiencies are not directly comparable across studies due to differences in chamber volume, pollutant loading, ventilation conditions, greening density, and exposure duration

| **Reference** | **iGI Type** | **CO_2_** | **VOC** | **TVOC** | ***Potential Negative Effect*** |
| --- | --- | --- | --- | --- | --- |
| Zhao et al. [51] | Potted Plants | - | HCHO removal: 129.88 mg FW. | - | Oxidative stress and transient accumulation of HCHO in plant tissues, potentially inhibiting physiology. |
| Bhargava et al. [52] | Potted plant | CO_2_ ↓20.13 ↓21.75 ↓52.33 ↓45.71 ↓10.48 |  | site I (floriculture laboratory), site II (chemistry laboratory), site III (canteen), and site IV (library) Outdoor  TVOC ↓66.01 ↓75.86 ↓83.04 ↓88.16 ↓57.69 | *Slight CO increases recorded at specific lab/canteen sites as plant numbers increased.* |
| Weerasinghe et al. [53] | Potted plant | Across four lighting levels (500–2000 lux),  Spathiphyllum blandum (+392%)  Aglaonema commutatum (+193%),  Philodendron hederaceum (+307%),  Chlorophytum comosum (+171%),  Dracaena fragrans (+147%) |  |  | CAM plants (Snake plant) emit during the day regardless of light intensity. |
| Teiri et al. [38] | Potted plant | - | ↓65–100%  81% under illumination vs. 59% in darkness | - | Capacity saturation occurs; removal efficiency drops when HCHO exceeds 12 . |
| Treesubsuntorn & Thiravetyan [54] | Potted plant | - | ↓15-20% | - | CO_2_ Net emission under light intensities of 10–50 mole PAR. |
| Irga et al. [40] | Potted plant | 37%± 3.1 of 1000 ppmv CO_2_ over a 40 min | - | - | - |
| Li et al. [55] | Potted plant |  | Chlorophytum Comosum    CC (green and white)  GC (Green)  PC (Purple)  Day  ↓85%,↓71%,  ↓47%  Night  ↓62%, ↓36%, ↓35% |  | Severe physiological damage (chlorophyll loss) under high concentrations (8 ppm) of HCHO. |
| Li et al. [56] | Potted plant | - | Day  ↓89.40% -48.78%    Night  ↓77.36% -25.40% | - | Irreversible stomatal damage from severe exposure leads to 35–50% loss in future air-cleaning efficiency. |
| Aini Jasmin et al. [57] | Potted plant | - | Nephrolepis exaltata ↓70% to 77%  Dracaena fragrans ↓63% to 72%  in Rhapis excelsa ↓75%to 81%, with the highest  value recorded in 6 plants. | - | Ineffectiveness in real rooms where high air exchange or ventilation masks the greening effect. |
| Dela Cruz et al. [44] | Potted plant | - | the plants achieved a removal rate of  66.5 μg/m2/h, whereas the removal rate was 28.7 μg/m2/h  under semi-dynamic conditions | - | - |
| Wood et al. [30] | Potted plant | - | 50–75% | - | Slight TVOC increase during the flowering phase of species like S. ‘Sweet Chico’. |
| Lin et al. [41] | Potted plant | - | Hedera helix can reduce 70% of the required time to reach 0.5 ppm of gaseous HCHO when compared with natural dissipation | - | Potential hazard risk from biogenic toxins (VOCs) emitted by the plants themselves. |
| Kim et al. [58] | Potted plant | - | Formaldehyde removal µgm-3cm-2 for 86 species. | - | - |
| Hörmann et al. [42] | Potted plant | - | Removal rate 1 to 9.5 mg/m3 | - | - |
| Berger et al. [59] | Potted plant | - | - | - | Risk of mold growth and condensation if plant transpiration keeps avg. RH above 70%. |
| *Orwell et al. [60]* | *Potted plant* | *-* | *Removal: 1014 mg for 100 ppm Toluene.* | *-* | *Toxic inhibition or saturation of removal rates observed at extreme (100 ppm) concentrations.* |
| Shen et al. [27] | Green wall | - | Reduce VOC concentration in 48h  Davallia fejeensis−41.27%; Maranta leuconeura−7.15%; Dryopteris erythrosora −55.85%; Adiantum sp. −38.25%; Philodendron hederaceum ‘Brasil’−2.58%  Increase VOC concentration in 48h  Platycerium bifurcatum +52.71%; Aglaonema 'Maria Christina' +41.49%; Philodendron micans +38.18%; Spathiphyllum wallisii +10.06%; Pachira aquática +82.21%; Calathea lancifolia +43.23%; Philodendron scandens +9.32% | - | Specific species (Rabbit Foot Fern) were observed to contribute to TVOC increases over time. |
| Matheson et al. [61] | Green wall | - | ↓42.47 ± 2.57 | - | - |
| Yang et al. [46] | Green wall | ↓2.45 | ↓53.8% | - | - |
| Helman et al. [62] | Green wall | measured intercellular CO_2_ | - | - | - |
| Dominici et al. [1] | Green wall | ↓1.68–7.95% | - | - | Suboptimal lighting leads to net-zero removal or CO_2_ increase. |
| Yungstein & Helman [2] | Green wall | ↓4.8%, | - | - | - |
| Tudiwer & Korjenic [28] | Green wall | ↓3.5% | - | - | Study specifically disproved mould fears |
| Shao et al. [25] | Green wall | ↓12% to 17% | - | - | - |
| Alsadun et al. [24] | Hydroponic | 14.1% ↓ | 28.1% ↓ | - |  |
| Irga et al. [40] | Hydroponic | ↓61% ± 2.2 of 1000 ppmv CO_2_ over a 40 min | - | - | Net CO_2_ emissions at low light intensities (≈10 µmol PAR m⁻² s⁻¹) due to plant/microbial respiration. |
| Aydogan & Montoya [63] | Hydroponic | Hedera Helix  ↓88 ± 2  Chrysanthemum morifolium  ↓84 ±1  Dieffenbachia compacta  ↓96±0  Epipremnum aurenum  ↓94 ± 2 | - | - | - |
| Caron et al. [29] | Biofilter | - | Toluene (↓94%), Decane (↓82%), Limonene (↓40%), Formaldehyde (↓47%), Acetaldehyde (↓96%), Propanal (↓85%), Acrolein (↓50%), Pentanal (↓67%), Hexanal (↓80%), Benzaldehyd (↓80%), Acetone (↓95%), Butan-2-one (↓68%) | - | Significant humidity increase (20% avg) interfering with the accuracy and performance of electronic gas sensors. |
| Ding et al. [35] | - | ↓9.3% | - | - | - |
| Torpy et al. [64] | - | - | 56.6 ± 0.86% | - | Potential for VOC saturation in substrate (activated carbon) over long periods; performance constrained by lighting needs. |

**Table S8.** Summary of PM and PTE removal associated with iGI. Reported removal efficiencies are not directly comparable across studies due to differences in chamber volume, pollutant loading, ventilation conditions, greening density, and exposure duration

| **Reference** | **iGI Type** | **PM_2.5_** | **PM_10_** | **PTE** | **Potential Negative Effect** |
| --- | --- | --- | --- | --- | --- |
| Yang et al. [46] | Green wall | ↓16.5% | ↓15.8%. | - | - |
| Ghazalli et al. [39] | Green wall | −48.5%, | −82.6%,(>PM₁₀: ↓5.5%) |  | The installation process can disperse planting media fragments into the air; manual leaf washing is required as there is no natural rain to remove dust |
| Iddio et al. [34] | Green wall | Higher thermal sensation when CO_2_ increase | - | - | - |
| Shao et al. [25] | Green wall | - | ↓8% to 14% | - | - |
| Kazemi et al. [45] | Hydroponic | Quantitative outcomes reported only for plant growth | - | - | Environmental contamination from substrate dust and increased salinity/EC from vermicompost use. |
| Alsadun et al. [24] | Hydroponic | 20.9% ↓ |  | - | Risk of humidity reaching 100% in minutes in unventilated spaces; presence of air backflow in modules |
| Rossini-Oliva et al. [65] | Biofilter | - | - | Potentially toxic element concentration carried by PM  ↓64–78.6%.  Tradescantia zebrina ↓ 65.4%; Philodendron scandens ↓ 72.3%; Ficus pumila ↓ 77.3%; Chlorophytum comosum ↓ 78.6% | Substantial increase in water requirements and energy consumption for continuous active operation |
| Irga et al. [66] | Biofilter | ↓48.21 ± 14.71% | ↓53.51 ± 15.99% |  | Removal efficiency declines at airflow rates above 11.25 L/s; increased power draw by fans to maintain flow. |
| Pettit et al. [43] | Biofilter | PM_0.3-0.5_ (45.78%) and PM_5-10_ (92.46%). | | - | Higher removal efficiencies are associated with increased pressure drop across the biofilter. |

**Table S9**. Summary of selected studies on outdoor–indoor greening interactions and IEQ impacts, highlighting study method, outdoor-to-indoor pathways, and their quantitative effects.

| **Study / location** | **Greening configuration (outdoor/indoor)** | **Scale & method** | **Primary IEQ domains** | **Outdoor to indoor mechanism** | **Key quantitative IEQ outcomes** |
| --- | --- | --- | --- | --- | --- |
| Rugani et al. [67], Rome | Evergreen green façade; no indoor greening | Façade and single office zone; multi-year monitoring and grey-box modelling | Thermal, heat–moisture | Vegetation and cavity lower sol-air temperature and damp thermal wave, reducing heat flux into various wall types | Heat flux −35–48%; wall amplitude −24–39%; peak indoor temperature up to 5.2°C lower in lightweight walls |
| Pastore et al. [68], Palermo | Neighbourhood trees, grass, hedges, living walls, green roof; no indoor plants | Neighbourhood + apartments; ENVI-met microclimate coupled with EnergyPlus | Thermal, energy | Outdoor vegetation cools façades and courtyards; modified microclimate used as boundary conditions for free-running apartments | Up to ~4.8°C indoor peak cooling; strong increase in adaptive comfort hours for exposed, top-floor dwellings |
| Taleghani et al. [69], Manchester | Existing park, river; additional street trees; no indoor greening | Field UHI measurements; ENVI-met and DesignBuilder/EnergyPlus coupling | Thermal, comfort | Added trees cool campus microclimate; local outdoor temperatures drive calibrated dwelling model under heatwave conditions | Park up to 3.6°C cooler than parking; extra trees reduce indoor temperature ~2.9°C and discomfort hours by ~21% on hottest day |
| Lei et al. [70], Guangzhou | Vertical green façade on west wall; no indoor greening | Paired rooftop test rooms; full-scale monitoring plus canopy heat-balance analysis | Thermal, comfort, energy | Canopy shading, transpiration and wind barrier change façade radiation and convection, altering wall heat flux and indoor conditions | External wall up to 14.2°C cooler; indoor OT peaks 3.6°C lower; AC electricity ~17% lower over hot days |
| Hao et al. [71], Xiangtan | Green roof and four green façades; no indoor plants | Twin rooftop rooms; field experiment and validated EnergyPlus model | Thermal, energy, comfort | Vegetated envelope reduces solar gains and surface temperatures, lowering mean radiant temperature and operative temperature at fixed air setpoints | Daytime OT ~0.6°C lower; operative swing −1.1°C; equivalent setpoint increase ~0.8°C saves ~7–8% cooling energy |
| Dadvand et al. [72], Barcelona | Neighbourhood greenness (NDVI); indoor plants not measured | Residential cohort; personal, indoor and outdoor monitoring with regression models | Air pollutants, exposure | Surrounding vegetation lowers ambient PM₂.₅ near homes and modestly changes time–activity, reducing indoor and personal exposure | IQR NDVI increase associated with ~6 μg/m³ lower home-indoor PM₂.₅ and ~5.9 μg/m³ lower personal PM₂.₅ |
| Matthaios et al. [73], NE USA schools | Urban green space (NDVI), road distance; no indoor greening | 74 schools; week-long indoor TRAP monitoring and mixed-effects modelling | Air pollutants, exposure | Greenness and distance from major roads reduce local outdoor TRAP; lower infiltrating load determines classroom concentrations | High-greenness distant schools show >60% lower indoor traffic-related PM₂.₅ and sizeable NO₂, BC reductions versus near-road low-greenness schools |
| Yang et al. [46], Changzhou | External VGS on façades; indoor living wall and potted plants | Full building; 90-day IEQ monitoring and annual energy analysis | Thermal, humidity, IAQ, energy | External VGS moderates indoor temperature response; indoor VGS plus plants act as sinks for PM and formaldehyde | PM₂.₅ and PM₁₀ reduced ~16–21%; CH₂O roughly halved; indoor temperature swings and RH variability significantly reduced |
| Azkorra et al. [74], Spain | Modular living wall on façade; no indoor greening | Laboratory transmission and reverberation room tests | Noise, acoustics | Vegetated panel and porous substrate increase sound absorption and transmission loss at the façade boundary | Weighted absorption αw ≈ 0.40; Rw ≈ 15–18 dB; reverberation times reduced by ~4–6 s in low-mid frequencies |
| Tang et al. [75], Nanjing | Climbing green wall and Sedum paving green wall; no indoor plants | Façade-scale field thermal and noise measurements on two buildings | Thermal, noise | Shading, evapotranspiration and soil evaporation cool surfaces; foliage and soil layers absorb and scatter sound through the wall | Paving green wall lowers surface temperature up to ~6–7°C (irrigated) and adds several dB noise reduction compared with bare wall |
| Zhang et al. [10], Shanghai | NDVI within 300 m, perceived community greenery; indoor plants, gardening, digital nature, green views | 386 adults; cross-sectional survey with regression and SEM | Psycho-physiological | Indoor and outdoor greenery increase perceived home restorativeness, reducing loneliness and fear, thereby lowering depressive symptoms | Higher greenery exposures associated with fewer depressive symptoms and reduced odds of PHQ-9 ≥10; pathways largely mediated by restorativeness, loneliness and fear |
| Spano et al. [11], Italy | Access to private green, greener road environment; indoor plant pots, green view, sunlight | Nationwide online survey; single-exposure regression models | Psycho-physiological | Indoor plants, daylight and views, plus private outdoor green, buffer emotional impacts of lockdown on residents | Presence of plants, good sunlight and green views associated with smaller increases in anxiety, irritability, boredom and sleep disturbance during lockdown |
| Dzhambov et al. [12], Plovdiv | Neighbourhood greenness, garden access; indoor houseplants and green window views | Students; online survey with SEM | Psycho-physiological | Indoor greenery improves home restorativeness; outdoor greenery improves neighbourhood restorativeness, social support and engagement, together reducing distress | More indoor plants and greener neighbourhoods linked with lower depression and anxiety scores; models require both indoor and outdoor greenery pathways for best fit |
| Paniccià et al. [13], Europe-focused review | Outdoor parks, trees, urban forests; indoor vegetation in buildings | Narrative health review synthesising multi-disciplinary evidence | Health, psycho-physiological | Ecosystem services from outdoor greening support cooler, cleaner environments; indoor vegetation and views provide direct restorative and comfort benefits | Greenness associated with reduced stress, better cognition; indoor plants alleviate sick-building symptoms and improve perceived comfort |

**Table S10.** Strategies to improve the efficiency of monitoring and modelling for climate-change research. The table summarises key challenges in climate observation and predictive modelling, integrated approaches to address them, and the expected benefits of improved coordination for accuracy and robustness in climate assessments.

| **Challenge** | **Recommended approach (monitoring + modelling)** | **Expected benefit** |
| --- | --- | --- |
| Changing outdoor climate envelopes (temperature, humidity, pollutants) | Use future weather data + holistic IAQ–IEQ models (emissions, reactions, mould, moisture). | Provides a unified platform to forecast how plant‑mediated cooling, moisture, and IAQ will perform under future climates. |
| Real‑time plant physiological response | Deploy IoT sensors on leaf temperature, substrate moisture, and sap flow; feed data into a plant‑transpiration model that updates the evapotranspiration rate in the building‑energy simulation (EnergyPlus, DesignBuilder). | Adaptive control that sustains cooling efficiency while preventing humidity overshoot. |
| Integrated IAQ‑IEQ outcomes | Extend IEQ indices to include vegetated surface contributions; use Bayesian calibration to align simulations with sensor data. | Comparable IEQ performance scores across climates and seasons. |
| Unintended consequences (e.g., mould, VOC re‑emission) | Integrate moisture balance + mould models (e.g., ASHRAE 160) and a VOC adsorption‑desorption model. | Early warning of moisture or pollutant hazards enabling proactive interventions. |
| Data‑driven model validation across climates | Run long‑term (≥ 1 yr) field pilots in at least three climate zones (hot‑arid, hot‑humid, temperate) and use the collected temperature, RH, pollutants and plant‑health data to train machine‑learning models. | Reduced reliance on heavy simulations, faster early-design guidance. |
| Lack of standardised IEQ monitoring protocols | Use a minimum sensor set (temp, RH, pollutants, bVOCs irrigation/energy use, etc.) with scheduled calibration. | High-quality comparable datasets enabling meta-analysis and rating-system integration. |

**References**

[1] L. Dominici, R. Fleck, R.L. Gill, T.J. Pettit, P.J. Irga, E. Comino, F.R. Torpy, Analysis of lighting conditions of indoor living walls: Effects on CO₂ removal, J. Build. Eng. 44 (2021) 102961.

[2] Y. Yungstein, D. Helman, Cooling, CO₂ reduction, and energy‑saving benefits of a green‑living wall in an actual workplace, Build. Environ. 236 (2023) 110220.

[3] F. Scamoni, C. Scrosati, M. Depalma, B. Barozzi, Experimental evaluations of acoustic properties and long‑term analysis of a novel indoor living wall, J. Build. Eng. 47 (2022) 103890.

[4] R. Jafar, W. Jabi, S. Lannon, Developing a digital workflow to estimate green walls’ performance for enhancing indoor air quality, in: Proceedings of Building Simulation 2023: 18th Conference of IBPSA, 2023, pp. 1741–1747. https://doi.org/10.26868/25222708.2023.1367

[5] B.B. Kanbur, G.K.K. Raveendran, S. Dubey, P.B. Rajesh, Plant-based Green Wall in Office Environment-Part 2: Steady-State Numerical Simulations, Energy Proc. 24 (2021), Paper 921. https://doi.org/10.46855/energy-proceedings-9729

[6] V. Mileikovskyi, T. Tkachenko, L. Kotelkov, Theoretical simulation of natural air exchange and indoor air quality with an example of a green wall introduction, Results Eng. 25 (2025) 104336.

[7] D. Zhang, L. Zhang, Y. Zhang, Investigation of the indoor CO_2_ removal efficiency and fresh air energy savings of living walls in office spaces, J. Build. Eng. 90 (2024) 109422. https://doi.org/10.1016/j.jobe.2024.109422

[8] N. Vivekanandan, K. Rajeswari, N.V. Yuvraj Kanna, A CFD-based assessment of Azolla biofilters for sustainable indoor air purification in low-carbon buildings, Int. J. Interact. Des. Manuf. 20 (2026) 285–298. https://doi.org/10.1007/s12008-025-02377-5

[9] N. Vivekanandan, K. Rajeswari, A coupled ODE–CFD framework for size-resolved modeling of indoor airborne microplastic mitigation with Azolla biofilters, Build. Environ. 287 (2026) 113867. https://doi.org/10.1016/j.buildenv.2025.113867

[10] J. Zhang, M.H.E.M. Browning, J. Liu, Y. Cheng, B. Zhao, P. Dadvand, Is indoor and outdoor greenery associated with fewer depressive symptoms during COVID-19 lockdowns? A mechanistic study in Shanghai, China, Build. Environ. 227 (2023) 109799. https://doi.org/10.1016/j.buildenv.2022.109799

[11] G. Spano, M. D’Este, V. Giannico, M. Elia, R. Cassibba, R. Lafortezza, G. Sanesi, Indoor–outdoor green features and psychological health during COVID-19 lockdown, Urban For. Urban Green. 62 (2021) 127156.

[12] A.M. Dzhambov, P. Lercher, M.H.E.M. Browning, D. Stoyanov, N. Petrova, S. Novakov, D.D. Dimitrova, Does greenery experienced indoors and outdoors provide an escape and support mental health during the COVID-19 quarantine?, Environ. Res. 196 (2021) 110420. https://doi.org/10.1016/j.envres.2020.110420

[13] M. Paniccià, M. Acito, I. Grappasonni, How outdoor and indoor green spaces affect human health: a literature review, Ann. Ig. 37(3) (2025) 333–349.

[14] P. Kumar, H. Sun, A. Biswal, et al., Ten questions on indoor greening and environmental quality, Build. Environ. 295 (2026) 114336.

[15] H. Rasheed, T.K. Jayasree, The multifaceted role of indoor plants: A comprehensive review of their impact on air quality, health, and perception, Energy Build. 330 (2025) 115312.

[16] F. Fonseca, M. Paschoalino, L. Silva, Health and well-being benefits of outdoor and indoor vertical greening systems: A review, Sustainability 15 (2023) 4107.

[17] M.S. Montaluisa-Mantilla, P. García-Encina, R. Lebrero, R. Muñoz, Botanical filters for the abatement of indoor air pollutants, Chemosphere 345 (2023) 140483. https://doi.org/10.1016/j.chemosphere.2023.140483

[18] F. Liu, L. Yan, X. Meng, C. Zhang, A review on indoor green plants employed to improve indoor environment, J. Build. Eng. 53 (2022) 104542.

[19] K.T. Han, L.W. Ruan, Effects of indoor plants on air quality: A systematic review, Environ. Sci. Pollut. Res. 27 (2020) 16019–16051.

[20] K. Gunawardena, K. Steemers, Living walls in indoor environments, Build. Environ. 148 (2019) 478–487. https://doi.org/10.1016/j.buildenv.2018.11.014

[21] L. Deng, Q. Deng, The basic roles of indoor plants in human health and comfort, Environ. Sci. Pollut. Res. 25(36) (2018) 36087–36101.

[22] T. Armijos Moya, A. van den Dobbelsteen, M. Ottelé, P.M. Bluyssen, A review of green systems within the indoor environment, Indoor Built Environ. 28(3) (2019) 298–309. https://doi.org/10.1177/1420326X18783042

[23] M. Dela Cruz, J.H. Christensen, J.D. Thomsen, R. Müller, Can ornamental potted plants remove volatile organic compounds from indoor air?—a review, Environ. Sci. Pollut. Res. 21(24) (2014) 13909–13928. https://doi.org/10.1007/s11356-014-3240-x

[24] I.S.R. Alsadun, F.M. Bashir, Z. Andleeb, Z. Ben Houria, M.A.S. Mohamed, O. Agboola, Synergistic Air Quality and Cooling Efficiency in Office Space with Indoor Green Walls, Buildings 15(20) (2025) 3656.

[25] Y. Shao, J. Li, Z. Zhou, F. Zhang, Y. Cui, The impact of indoor living wall system on air quality: a comparative monitoring test in building corridors, Sustainability 13(14) (2021) 7884.

[26] F. Salamone, B. Barozzi, L. Danza, M. Ghellere, I. Meroni, Correlation between Indoor Environmental Data and Biometric Parameters for the Impact Assessment of a Living Wall in a ZEB Lab, Sensors 20(9) (2020) 2523. https://doi.org/10.3390/s20092523

[27] X. Shen, Q. Sun, G. Mosey, J. Ma, L. Wang, M. Ge, Benchmark of plant-based VOCs control effect for indoor air quality: Green wall case in smith campus at Harvard University, Sci. Total Environ. 906 (2024) 166269. https://doi.org/10.1016/j.scitotenv.2023.166269

[28] D. Tudiwer, A. Korjenic, The effect of an indoor living wall system on humidity, mould spores and CO₂ concentration, Energy Build. 146 (2017) 73–86.

[29] A. Caron, N. Redon, F. Thevenet, B. Hanoune, P. Coddeville, Performances and limitations of electronic gas sensors to investigate an indoor air quality event, Build. Environ. 107 (2016) 19–28.

[30] R.A. Wood, M.D. Burchett, R. Alquezar, R.L. Orwell, J. Tarran, F. Torpy, The potted-plant microcosm substantially reduces indoor air VOC pollution: I. Office field-study, Water Air Soil Pollut. 175(1) (2006) 163–180.

[31] Y. Shao, J. Li, Z. Zhou, Z. Hu, F. Zhang, Y. Cui, H. Chen, The effects of vertical farming on indoor carbon dioxide concentration and fresh air energy consumption in office buildings, Build. Environ. 195 (2021) 107766. https://doi.org/10.1016/j.buildenv.2021.107766

[32] Y.-M. Su, C.-H. Lin, Removal of Indoor Carbon Dioxide and Formaldehyde Using Green Walls by Bird Nest Fern, Hortic. J. 84(1) (2015) 69–76. https://doi.org/10.2503/hortj.CH-114

[33] Z. Poorova, Z. Vranayova, Humidity, Air Temperature, CO₂ and Well-Being of People with and Without Green Wall, in: Z. Blikharskyy (Ed.), Proceedings of EcoComfort 2020, Lecture Notes in Civil Engineering, vol. 100, Springer, Cham, 2021, pp. 336–346. https://doi.org/10.1007/978-3-030-57340-9_41

[34] E. Iddio, L. Wang, H. Zhang, N.H. Wong, The effects of indoor living walls on occupant thermal comfort in office buildings, Sci. Technol. Built Environ. 31(4) (2025) 398–414. https://doi.org/10.1080/23744731.2025.2460371

[35] N. Ding, F. Liu, F. Pang, J. Su, L. Yan, X. Meng, Integrating the living wall with the split air conditioner towards indoor heating environment improvement in winter, Case Stud. Therm. Eng. 47 (2023) 103061.

[36] X. Shen, Q. Sun, G. Mosey, Y. Cai, W. Cheng, Optimizing indoor air quality: The role of potted plants and machine learning models in reducing VOCs, Urban Clim. 62 (2025) 102497. https://doi.org/10.1016/j.uclim.2025.102497

[37] S. Abedi, S. Soleimani‑Alyar, B. Shahi‑bayatany, A. Jafari Joneidi, R. Yarahmadi, Thermal effects, experimental evaluation of evaporative cooling performance and energy efficiency of active botanical biofilter for indoor air treatment, J. Build. Eng. 108 (2025) 112950.

[38] H. Teiri, H. Pourzamani, Y. Hajizadeh, Phytoremediation of VOCs from indoor air by ornamental potted plants: A pilot study using a palm species under the controlled environment, Chemosphere 197 (2018) 375–381. https://doi.org/10.1016/j.chemosphere.2018.01.078

[39] A.J. Ghazalli, C. Brack, X. Bai, I. Said, Alterations in use of space, air quality, temperature and humidity by the presence of vertical greenery system in a building corridor, Urban For. Urban Green. 32 (2018) 177–184.

[40] P.J. Irga, F.R. Torpy, M.D. Burchett, Can hydroculture be used to enhance the performance of indoor plants for the removal of air pollutants?, Atmos. Environ. 77 (2013) 267–271.

[41] M.W. Lin, L.Y. Chen, Y.K. Chuah, Investigation of a potted plant (Hedera helix) with photo-regulation to remove volatile formaldehyde for improving indoor air quality, Aerosol Air Qual. Res. 17(10) (2017) 2543–2554.

[42] V. Hörmann, K.R. Brenske, C. Ulrichs, Suitability of test chambers for analyzing air pollutant removal by plants and assessing potential indoor air purification, Water Air Soil Pollut. 228(10) (2017) 402.

[43] T. Pettit, P.J. Irga, P. Abdo, F.R. Torpy, Do the plants in functional green walls contribute to their ability to filter particulate matter?, Build. Environ. 125 (2017) 299–307.

[44] M. Dela Cruz, R. Müller, B. Svensmark, J.S. Pedersen, J.H. Christensen, Assessment of volatile organic compound removal by indoor plants—A novel experimental setup, Environ. Sci. Pollut. Res. 21 (2014) 7838–7846.

[45] F. Kazemi, M. Rabbani, M. Jozay, Investigating the plant and air‑quality performances of an internal green wall system under hydroponic conditions, J. Environ. Manag. 275 (2020) 111230.

[46] Y. Yang, K. Hu, Y. Liu, Z. Wang, K. Dong, P. Lv, X. Shi, Optimisation of building green performances using vertical greening systems: A case study in Changzhou, China, Sustainability 15(5) (2023) 4494.

[47] L. Pérez‑Urrestarazu, R. Fernández‑Cañero, A. Franco, G. Egea, Influence of an active living wall on indoor temperature and humidity conditions, Ecol. Eng. 90 (2016) 120–124.

[48] K.R. Gunawardena, K. Steemers, Living wall influence on microclimates: An indoor case study, J. Phys. Conf. Ser. 1343 (2019) 012188. https://doi.org/10.1088/1742-6596/1343/1/012188

[49] G. Qadir, N. Wijesooriya, A. Brambilla, F. Alonso-Marroquin, Improving the Indoor Environment through an Indoor Green Curtain System, Buildings 13(5) (2023) 1307.

[50] P. Abdo, B.P. Huynh, An experimental investigation of green wall bio‑filter towards air temperature and humidity variation, J. Build. Eng. 39 (2021) 102244.

[51] S. Zhao, Y. Su, H. Liang, Efficiency and mechanism of formaldehyde removal from air by two wild plants: Plantago asiatica L. and Taraxacum mongolicum Hand.-Mazz, J. Environ. Health Sci. Eng. 17 (2019) 141–150.

[52] B. Bhargava, S. Malhotra, A. Chandel, A. Rakwal, R.R. Kashwap, S. Kumar, Mitigation of indoor air pollutants using Areca palm potted plants in real-life settings, Environ. Sci. Pollut. Res. 28 (2021) 8898–8906. https://doi.org/10.1007/s11356-020-11177-1

[53] N.H. Weerasinghe, P.K. Silva, R.R. Jayasinghe, W.P. Abeyrathna, G.K.P. John, R.U. Halwatura, Reducing CO₂ level in the indoor urban built environment: Analysing indoor plants under different light levels, Clean. Eng. Technol. 14 (2023) 100645.

[54] C. Treesubsuntorn, P. Thiravetyan, Botanical biofilter for indoor toluene removal and reduction of carbon dioxide emission under low light intensity by using mixed C3 and CAM plants, J. Clean. Prod. 194 (2018) 94–100.

[55] J. Li, J. Zhong, T. Zhan, et al., Indoor formaldehyde removal by three species of Chlorophytum comosum under a long-term dynamic fumigation system, Environ. Sci. Pollut. Res. 26 (2019) 36857–36868.

[56] J. Li, J. Zhong, Q. Liu, H. Yang, W. Zhiyu, Y. Li, W. Zhang, I.E. Agranovski, Indoor formaldehyde removal by three species of Chlorophytum comosum under dynamic fumigation system: Part 2—Plant recovery, Environ. Sci. Pollut. Res. 28 (2021) 8453–8465.

[57] G. Aini Jasmin, M. Noorizan, M. Suhardi, A.G. Murad, K. Ina, The use of plants to improve indoor air quality in small office space, Pertanika J. Soc. Sci. Humanit. 20(2) (2012) 493–503.

[58] K.J. Kim, M.I. Jeong, D.W. Lee, J.S. Song, H.D. Kim, E.H. Yoo, S.J. Jeong, S.W. Han, S.J. Kays, Y.W. Lim, H.H. Kim, Variation in formaldehyde removal efficiency among indoor plant species, HortScience 45(10) (2010) 1489–1495.

[59] J. Berger, E. Essah, T. Blanusa, The impact of plants on the humidity of naturally‑ventilated office indoor environments, J. Build. Eng. 86 (2024) 108814.

[60] R.L. Orwell, R.A. Wood, M.D. Burchett, J. Tarran, F. Torpy, The potted-plant microcosm substantially reduces indoor air VOC pollution: II. Laboratory study, Water Air Soil Pollut. 177(1) (2006) 59–80.

[61] S. Matheson, R. Fleck, T. Lockwood, R.L. Gill, P.J. Irga, F.R. Torpy, Fuelling phytoremediation: Gasoline degradation by green wall systems—A case study, Environ. Sci. Pollut. Res. 30(56) (2023) 118545–118555.

[62] D. Helman, Y. Yungstein, G. Mulero, Y. Michael, High-throughput remote sensing of vertical green living walls (VGWs) in workplaces, Remote Sens. 14(14) (2022) 3485.

[63] A. Aydogan, L.D. Montoya, Formaldehyde removal by common indoor plant species and various growing media, Atmos. Environ. 45(16) (2011) 2675–2682.

[64] F. Torpy, N. Clements, M. Pollinger, A. Dengel, I. Mulvihill, C. He, P. Irga, Testing the single-pass VOC removal efficiency of an active green wall using methyl ethyl ketone (MEK), Air Qual. Atmos. Health 11(2) (2018) 163–170.

[65] S. Rossini‑Oliva, J.M. Montiel de La Cruz, A.J. Fernández‑Espinosa, R. Fernández‑Cañero, V.M. Fernández‑Cabañas, L. Pérez Urrestarazu, Potentially toxic elements capture by an active living wall in indoor environments: Effect of species in air phytoremediation, Chemosphere 340 (2023) 139799.

[66] P.J. Irga, N.J. Paull, P. Abdo, F.R. Torpy, An assessment of the atmospheric particle removal efficiency of an in-room botanical biofilter system, Build. Environ. 115 (2017) 281–290.

[67] R. Rugani, G. Salvadori, C. Gargari, M. Picco, P. De Rossi, A. Latini, C. Bibbiani, F. Fantozzi, Assessing thermal performance of green walls for solar shading, Energy Build. 349 (2025) 116539.

[68] L. Pastore, R. Corrao, P.K. Heiselberg, Effects of vegetation on indoor thermal comfort: Multi-scale simulation, Energy Build. 146 (2017) 1–11.

[69] M. Taleghani, A. Marshall, R. Fitton, W. Swan, Impact of neighborhood greening on indoor thermal comfort during heatwaves, Sol. Energy 182 (2019) 245–255.

[70] Z. Lei, D. Zhichao, L. Lisha, Z. Yu, M. Qinglin, W. Junsong, M. Santamouris, Thermal behavior of a vertical green façade and its impact on thermal environment, Energy Build. 204 (2019) 109502.

[71] X. Hao, Q. Xing, P. Long, Y. Lin, J. Hu, H. Tan, Influence of vertical greenery systems and green roofs on indoor operative temperature, J. Build. Eng. 31 (2020) 101373.

[72] P. Dadvand, A. de Nazelle, M. Triguero-Mas, A. Schembari, M. Cirach, E. Amoly, F. Figueras, X. Basagaña, B. Ostro, M. Nieuwenhuijsen, Surrounding greenness and exposure to air pollution during pregnancy, Environ. Health Perspect. 120(9) (2012) 1286–1290.

[73] V.N. Matthaios, I. Holland, C.M. Kang, J.E. Hart, M. Hauptman, J.M. Wolfson, J.M. Gaffin, W. Phipatanakul, D.R. Gold, P. Koutrakis, The effects of urban green space and road proximity on indoor pollution, J. Expo. Sci. Environ. Epidemiol. 34 (2024) 745–752.

[74] Z. Azkorra, G. Pérez, J. Coma, L.F. Cabeza, S. Burés, J.E. Álvaro, A. Erkoreka, M. Urrestarazu, Evaluation of green walls as a passive acoustic insulation system for buildings, Appl. Acoust. 89 (2015) 46–56.

[75] V.T. Tang, E.R. Rene, L. Hu, S.K. Behera, N.T. Phong, C. Thi Da, Vertical green walls for noise and temperature reduction, Sci. Technol. Built Environ. 27(6) (2021) 806–818.
